# Near-Infrared Plasmonic Nanogap Organic Photodetectors for On-Chip Photonic Integration

**DOI:** 10.1021/acsnano.6c08504

**Published:** 2026-09-15

**Authors:** Danbi Kim, Hanna Lee, Changsoon Choi, Jung Pyo Hong, Do Kyung Hwang, Wassie M. Takele, Terefe G. Habteyes, Jeong Ho Cho, In−Ho Lee, Jae-Hoon Han, Jung Ah Lim

**Affiliations:** † Electronic and Hybrid Materials Research Center, 58975 Korea Institute of Science and Technology, Seoul 02792, Republic of Korea; ‡ Department of Chemical and Biomolecular Engineering, 26721 Yonsei University, Seoul 03722, Republic of Korea; § School of Electronic and Electrical Engineering, 543379 Sungkyunkwan University, Suwon 16419, Republic of Korea; ∥ Center for Quantum Technology, 58975 Korea Institute of Science and Technology, Seoul 02792, Republic of Korea; ⊥ KU-KIST Graduate School of Converging Science and Technology, Korea University, Seoul 02841, Republic of Korea; # Department of Chemistry and Chemical Biology, 1104 University of New Mexico, Albuquerque, New Mexico 87131, United States; ∇ GIST InnoCORE Al-Nano Convergence Institute for Early Detection of Neurodegenerative Diseases, Gwangju Institute of Science and Technology, Gwangju 61005, Republic of Korea; ○ Division of Nano and Information Technology, KIST School, University of Science and Technology (UST), Seoul 02792, Republic of Korea; ◆ Department of Materials Science and Engineering, YU-KIST Institute, Yonsei University, Seoul 03722, Republic of Korea

**Keywords:** organic photodetector, organic photodiode, near-infrared, integrated photonic circuit, plasmonic nanogap, plasmonic field enhancement, waveguide-integrated photodetector

## Abstract

Near-infrared (NIR) photonic integrated circuits (PICs) are emerging as key platforms for on-chip optical communication and sensing, driven by their compatibility with silicon photonics and low-loss operation in telecom wavelength bands. However, conventional photodetectors based on Si, Ge, and III–V semiconductors require high-temperature processing and are difficult to integrate with polymeric or low-index photonic systems. Organic photodetectors (OPDs) offer a promising alternative, enabling efficient NIR light–matter interaction and photodetection while remaining compatible with low-temperature, solution-processed fabrication. Here, we demonstrate a plasmonic nanogap OPD architecture that enables efficient photodetection within an extremely confined nanoscale channel and direct integration with few-micron-width waveguides. Au/Al nanogap electrodes generate strongly localized plasmonic fields and facilitate efficient carrier extraction. With a photomultiplication-type bulk heterojunction, the device achieves high responsivity and quantum efficiency, along with ultralow dark current and a fast temporal response. The waveguide-integrated device detects guided optical signals at multimode interference outputs with consistent modulation response.

## Graphical abstract

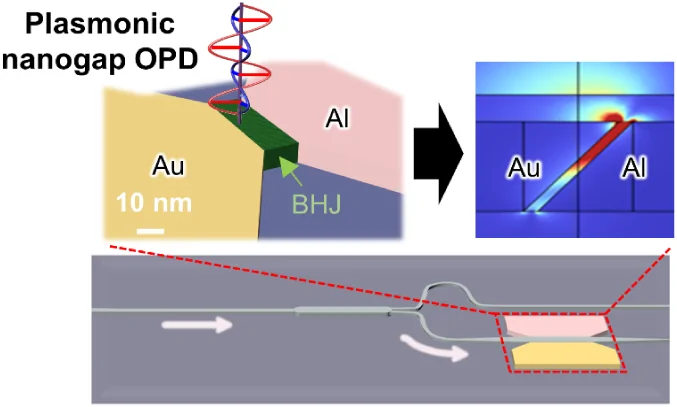

## Introduction

Photonic integrated circuits (PICs) are emerging as the foundation of on-chip optical technologies for communication, sensing, and signal processing.^1,2^ By routing and manipulating light within micrometer-scale waveguides, PICs provide a scalable, energy-efficient platform that bridges the photonic and electronic domains. Most photonic technologies operate in the near-infrared (NIR) or longer-wavelength (short-wavelength infrared, SWIR) regions (≈0.8–2.5 μm), where optical fibers, SiNx, SiO_2_, and Si-on-insulator (SOI) waveguides exhibit minimal propagation loss, and where compact and low-cost laser diodes, including vertical-cavity surface-emitting lasers (VCSELs) and distributed-feedback (DFB) sources, are readily available.^3,4^ Consequently, the ability to detect optical signals within this NIR spectral window is a prerequisite for practical on-chip photonic integration. However, conventional Si, Ge, and III–V semiconductor photodetectors rely on high-temperature epitaxy and remain difficult to integrate monolithically with polymeric or low-index photonic platforms. This limitation presents a critical bottleneck to realizing scalable and materials-diverse photonic systems.^5,6^

Organic photodetectors (OPDs) have emerged as a promising class of optoelectronic devices owing to their solution-processability, mechanical flexibility, and molecularly tunable optical properties.^7–9^ Advances in donor and acceptor organic semiconductor materials and device engineering have enabled OPDs to achieve detectivities and response speeds comparable to those of conventional silicon photodiodes.^10–12^ In particular, the development of nonfullerene acceptors and low-band gap polymers has extended their spectral sensitivity from the visible to the NIR and SWIR regions, enabling photodetection well beyond the intrinsic absorption limit of silicon.^10,13–15^ These attributes make OPDs attractive candidates for integrated photonic systems; however, their implementation within PIC architectures has remained largely unexplored.

Achieving on-chip compatibility in OPDs requires device geometries that can efficiently interact with guided optical fields rather than relying on normally incident illumination. Most reported OPDs employ multilayer, vertical sandwich-type geometries, in which light enters perpendicularly through a transparent electrode and interfacial charge-transport layers (e.g., hole- or electron-transport layers). Such configurations are inherently incompatible with planar photonic platforms, where optical signals propagate laterally through sub- to few-micrometer-wide waveguides. Efficient evanescent-field coupling from integrated waveguides typically requires a long interaction length between the photodetector and the optical mode, which limits device miniaturization and integration density.^16,17^ To address this limitation, waveguide-integrated photodetectors have employed coplanar geometries that enable direct coupling between the guided optical field and the photoactive material of the photodetector.^18–20^ In this context, waveguide-integrated graphene and other two-dimensional-material photodetectors have demonstrated efficient coupling of guided light to subsequent optical-to-electrical signal conversion recently.^21–23^ However, extending such architectures to OPDs while maintaining high responsivity and fast response in the NIR regime remains a significant challenge. Developing OPDs that can efficiently operate under guided-wave excitation would therefore represent a critical step toward monolithically integrated organic photonic systems.

Here, we demonstrate a plasmonic nanogap organic photodetector architecture that enables efficient photodetection within an extremely confined nanoscale channel while remaining directly compatible with planar photonic waveguides. The coplanar nanogap electrode geometry provides strong plasmonic field enhancement, overcoming the limited light–matter interaction typically associated with coplanar devices. By introducing photomultiplication (PM)-inducing polymeric photoactive materials into the nanogap channel via simple spin coating, the device achieves high responsivity, external quantum efficiency, and a high dynamic range in the near-infrared regime, comparable to those of state-of-the-art OPDs. A polymer waveguide deposited directly atop the photoactive channel enables efficient evanescent-field coupling, thereby converting guided optical signals into photocurrent at the waveguide-integrated photodetectors.

## Results and Discussion

Figure 1a schematically illustrates the device architecture of the plasmonic nanogap organic photodetectors (PN-OPDs). Asymmetric metal contacts consisting of gold (Au) and aluminum (Al) were patterned to form a nanoscale channel (nanogap) using an adhesion lithography technique, as previously reported.^24^ The schematic fabrication process is summarized in Figure S1 (Supporting Information), which outlines the sequential steps for forming nanogap electrodes and OPD arrays on silicon wafers or glass substrates. The nanogap distance is precisely defined by the molecular chain length of the self-assembled monolayer (SAM) formed on the Al surface, enabling uniform sub-20 nm nanogaps over a large area (Figure S2, Supporting Information), which are difficult to realize using conventional photolithography.^25^ A cross-sectional transmission electron microscopy (TEM) image in Figure 1b confirms the successful formation of the Au/Al nanogap structure. The slanted sidewall observed through the film depth originates from limited light penetration during photolithography with a positive photoresist on the Al layer.^26^ The average electrode spacing between Au and Al was approximately 10 nm (maximum ≈ 20 nm). Although a small number of Au nanoparticles were occasionally observed beneath the Au electrode and within the nanogap—likely due to partial penetration of Au into the underlying Al adhesion layer—the current mapping and electrical measurements (Figure 1c) confirm that the fabricated nanogaps are electrically isolated, ensuring complete disconnection between the two electrodes.

**Figure 1:**
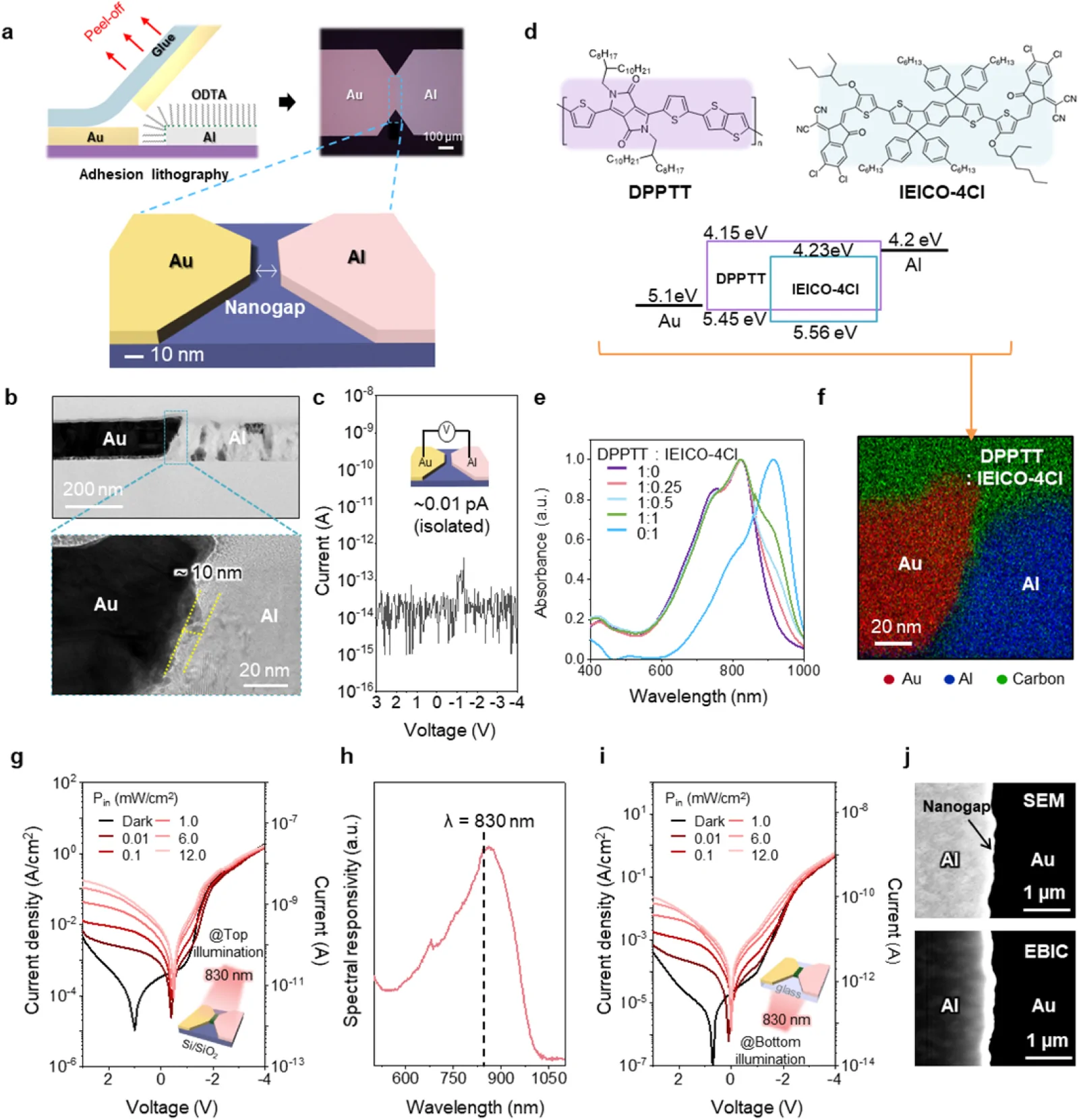
Nanogap device architecture and photoresponse of BHJ-filled PN-OPDs. (a) OM images and schematic device configuration of the plasmonic nanogap organic photodetectors (PN-OPDs). (b) Cross-sectional TEM image of a nanogap in PN-OPD. (c) Electrical measurement of the nanogaps confirming electrical isolation. (d) (Top) The chemical structures of the DPPTT and IEICO-4Cl materials used in the BHJ photoactive layer. (Bottom) Corresponding energy band diagram. (e) The optical absorption spectra of the BHJ films with different donor-to-acceptor ratios. (f) Cross-sectional TEM–EDS elemental mapping of the BHJ layer spin-coated on the PN-OPD. (g) Current–voltage (I–V) characteristics of the PN-OPD measured under dark conditions (black line) and under 830 nm top illumination with varying light intensities. (h) Spectral responsivity of the PN-OPD measured as a function of wavelength. (i) I–V characteristic of the PN-OPD fabricated on a glass substrate measured under bottom illumination with varying light intensities. (j) (Top) SEM image acquired in secondary electron (SE) mode showing the nanogap. (Bottom) EBIC image visualizing the localized current generation within the nanogap.

Efficient photodetection within the nanogap requires a photoactive material that uniformly fills the nanoconfined channel while maintaining continuous charge-transport pathways. To achieve this, we employed an organic bulk heterojunction (BHJ) system, which can be deposited from solution and provides donor–acceptor interfaces at the molecular scale for efficient exciton dissociation and charge collection. The photoactive layer consists of the low-bandgap polymer DPPTT and the ultralow-bandgap nonfullerene acceptor IEICO-4Cl, enabling near-infrared (NIR) operation^27^ (Figure 1d). As shown in Figure 1e, increasing the fraction of IEICO-4Cl enhances the absorption shoulder near 900 nm, consistent with increased acceptor aggregation, while the DPPTT-related shoulder around 750 nm decreases, indicating enhanced donor–acceptor mixing at the nanoscale.^28^ The DPPTT:IEICO-4Cl photoactive layer was spin-coated directly onto the nanogap electrodes, and cross-sectional elemental mapping analysis (Figure 1f) confirmed complete infiltration of the photoactive material into the nanogap channel.

The current–voltage (I–V) characteristics of the PN-OPD (Figure 1g) reveal clear rectifying behavior with an ultralow dark current (approximately 0.2 pA at 1 V), in contrast to the negligible current observed in empty nanogap electrodes. The temperature-dependent dark current measurements of the PN-OPD reveal barrier-mediated transport^29^ (Figure S3, Supporting Information), suggesting the presence of a Schottky barrier at the Al and BHJ interface. This barrier effectively suppresses carrier injection under dark conditions, contributing to the ultralow dark current. The positive onset voltage (∼1 V) is attributed to the built-in potential arising from the work function difference between the Au and Al electrodes, consistent with previously reported nanogap diode structures.^30^ Under 830 nm NIR illumination, photogenerated carriers partially screen the built-in potential and modify the effective injection barrier, resulting in a pronounced photocurrent and an open-circuit voltage that increases with light intensity from 0.01 to 12 mW cm^–2^. The spectral responsivity (Figure 1h) closely follows the absorption profile of the DPPTT:IEICO-4Cl photoactive layer, confirming that the photocurrent generation originates from optical excitation within the nanogap-embedded photoactive material.

To unambiguously identify the nanogap channel as the primary photoactive region, the device was measured under bottom illumination through the glass substrate (Figure 1i). Under this bottom-illumination geometry, the 100 nm-thick metal electrodes effectively block incident light from reaching the BHJ overlayer on the electrode regions, whereas the BHJ confined within the nanogap channel remains directly exposed to light through the glass substrate. Interestingly, the device showed a comparable photocurrent behavior under reverse bias for both top and bottom illumination, indicating that the dominant photoresponse originates from the nanogap channel rather than from the overlying film. Notably, despite the extremely small effective photoactive area of the PN-OPD (3 × 10^–8^ cm^2^, corresponding to 10 nm × 300 μm), the device generates a strong photocurrent, demonstrating that efficient photodetection can be achieved even within a highly confined nanoscale region.

To further substantiate the origin of photocurrent generation, electron-beam-induced current (EBIC) measurements were performed. In EBIC, local electron–hole pairs are generated by a focused electron beam, and the resulting current collected at the external electrodes visualizes the spatial distribution of charge collection efficiency.^31^ As shown in Figure 1j, a bright stripe observed along the nanogap region clearly evidences that carrier generation and extraction are confined within the nanoscale channel, while negligible signal is observed from the BHJ located on top of the Au and Al electrodes. In addition, we fabricated control devices with different residual BHJ widths remaining on the electrode regions (20, 50, and 80 μm), as shown in Figure S4 (Supporting Information). Because the entire device area was uniformly illuminated during the measurement, any significant photocurrent contribution from the overlying BHJ would be expected to increase with increasing overlayer area. However, the measured photocurrent remains almost unchanged despite a 4-fold variation in the residual BHJ width. This result indicates that the BHJ residing on the electrode regions makes a negligible contribution to the overall photocurrent. Moreover, increasing the BHJ thickness from 25 to 100 nm resulted in significant deterioration of the device photoresponse due to the substantial increase in dark current under top illumination (Figure S5, Supporting Information). This observation demonstrates that simply increasing the volume of the overlying BHJ does not enhance photocurrent generation, further supporting the conclusion that the nanogap-confined BHJ dominates the device photoresponse.

Interestingly, the plasmonic nanogap architecture provides a versatile photodetection platform for various organic photoactive materials with different photoactivation energies. Additional photoresponse results obtained from devices incorporating other donor–acceptor systems are provided in Figure S6 (Supporting Information). These devices also exhibited distinct photocurrent generation and low dark current, demonstrating that the nanogap coplanar design can be broadly applied across a range of organic semiconductor systems.

Although the nanogap channel is extremely small, the bulk-heterojunction (BHJ) structure plays a critical role in determining the photodetection performance of the device. Indeed, devices based on pristine DPPTT showed negligible photoresponse (Figure S7, Supporting Information), highlighting donor–acceptor heterojunctions within the nanogap. To evaluate the influence of DPPTT:IEICO-4Cl BHJ composition, the key performance metrics of the PN-OPD—responsivity (*R*), external quantum efficiency (EQE), and specific detectivity (D*)—were systematically analyzed for PN-OPDs fabricated with various DPPTT:IEICO-4Cl blend ratios (D:A ratios). The calculation procedures are described in the Methods section. As shown in Figure 2a, *R* decreases with increasing light intensity for all compositions, consistent with bimolecular recombination limiting carrier collection at high illumination levels.^32^ While the pristine DPPTT device showed negligible photoresponse, the BHJ device with a D:A ratio of 1:0.1 exhibited only the onset of photoresponse, with a responsivity of 0.006 A W^1–^ and an EQE of 6.87% (Figure S8, Supporting Information). Among the tested devices, the 1:0.25 D:A ratio exhibits the highest *R*, reaching ≈160 A W^1–^ at a light intensity of 0.012 mW cm^–2^. Notably, this device maintains *R* > 1 A W^1–^ even at strong illumination (12 mW cm^–2^), corresponding to EQE exceeding 100% due to the photogain effect (inset, Figure 2a). Consistently, the highest EQE is observed for the 1:0.25 device, while even the lowest EQE among all compositions remains above 500% at high illumination (Figure 2b and Figure S9, Supporting Information). The optimized device exhibits a D* of 6 × 10^9^ Jones, arising from enhanced *R* and ultralow dark current (Figure 2b, right *y*-axis).^33^

**Figure 2:**
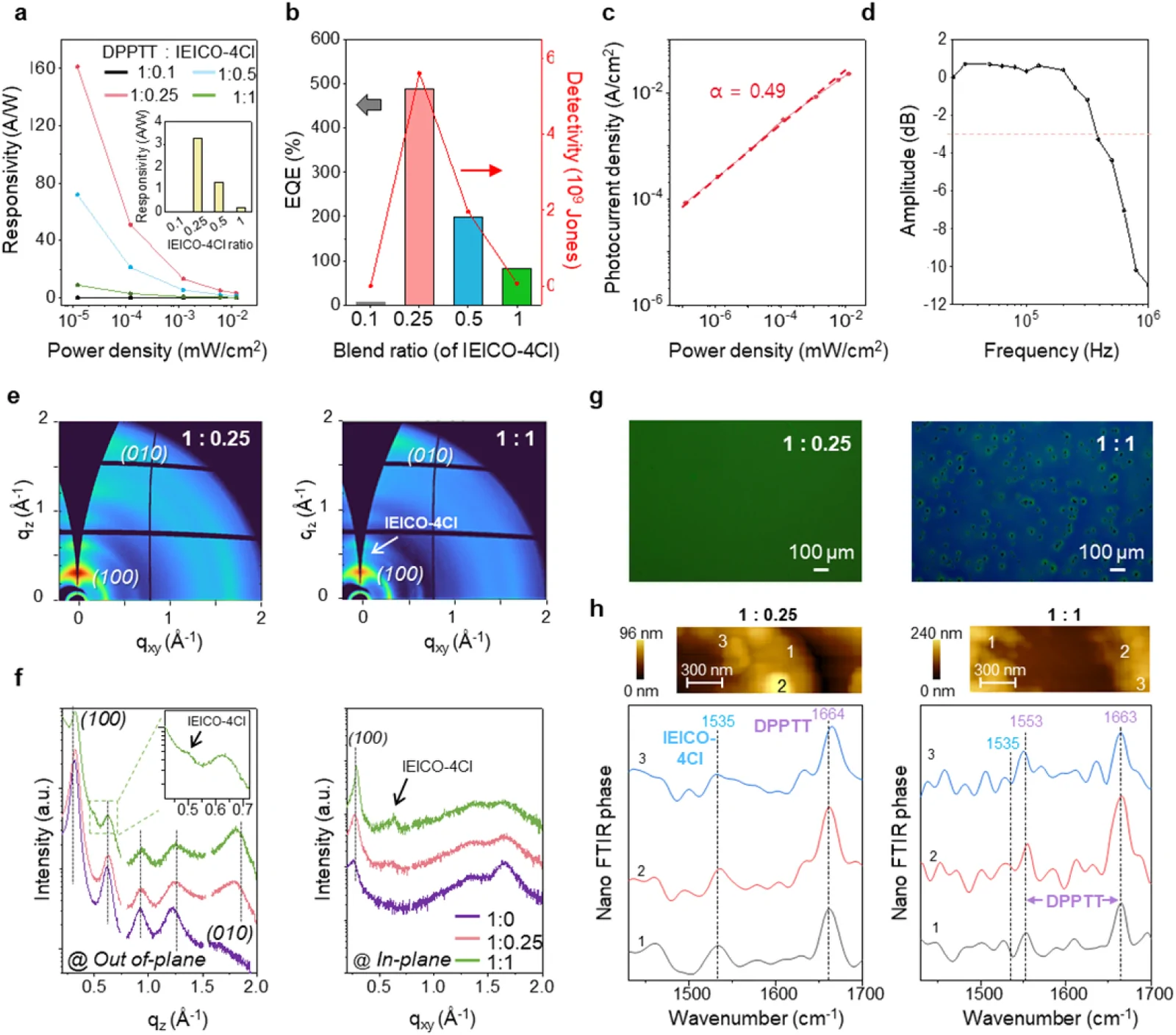
Photodetection performance and morphology of optimized BHJ in PN-OPDs. (a) *R* of the PN-OPDs measured at a bias of 1 V under varying light power densities for different D:A ratios. The inset presents *R* measured at the maximum light power density (2 mW cm^‑2^). (b) Corresponding EQE and D* values extracted at 2 mW cm^‑2^ under a 1 V bias, with the highest values observed for the 1:0.25 D:A ratio. (c) Photocurrent of the PN-OPD at the optimized D:A ratio (1:0.25) measured under varying light densities. (d) Frequency response of the 1:0.25 PN-OPD device. The normalized photocurrent as a function of modulation frequency measured under 852 nm illumination at a bias of 1 V. (e) 2D-GIXD pattern of the BHJ films with different blend ratios (1:0.25 and 1:1), showing diffraction peaks characteristic of the pristine IEICO-4Cl film in the 1:1 blend. (f) 1D-GIXD profiles of pristine DPPTT and BHJ films with D:A ratios of 1:0.25 and 1:1, showing the emergence of diffraction peaks associated with IEICO-4Cl only in the 1:1 blend, as highlighted in the inset. (g) OM image of the BHJ films with D:A ratios of 1:0.25 and 1:1, with visible phase separation observed in the 1:1 film. (h) s-SNOM combined with FT-IR nanospectroscopy of BHJ films, showing the coexistence of donor- and acceptor-related vibrational peaks within nanograins at the optimized ratio (1:0.25, left), while acceptor-related peaks are absent in the 1:1 blend (right).

The relationship between incident light power density and photocurrent density (Figure 2c) shows a continuous increase in photocurrent over the measured illumination range. The power-law exponent (α), extracted from the slope of double-logarithmic photocurrent-power plots, is ≈0.49 across the measured intensity range. This sublinear dependence (α value close to 0.5) indicates that the PN-OPD operates in a trap-assisted photomultiplication regime, consistent with the observed high EQE values.^34,35^ Furthermore, the transient photoresponse exhibits a rise time of 13.6 μs and a fall time of 9.5 μs (Figure S11, Supporting Information), confirming microsecond-scale switching of the PN-OPD. The frequency response measurements (Figure 2d) reveal a −3 dB bandwidth of 354 kHz. This relatively high bandwidth for a PM-type OPD demonstrates that substantial photogain can coexist with a fast temporal response in the nanogap architecture. A comprehensive comparison of the PN-OPD performance with representative NIR photomultiplication-type OPDs is provided in Table S1 (Supporting Information).

To elucidate the origin of the composition-dependent performance, the surface morphologies of BHJ films with different D:A ratios were examined using optical microscopy (OM) and atomic force microscopy (AFM). As shown in Figure 2e, the 1:1 D:A ratio exhibits pronounced microscopic phase separation, whereas the 1:0.25 D:A film maintains a uniform appearance without visible domains. AFM height profiles further show that surface roughness increases systematically with increasing IEICO-4Cl content (Figure S12, Supporting Information). These results indicate that increasing the IEICO-4Cl fraction promotes phase separation at the microscale, which is detrimental to forming a uniformly intermixed BHJ compatible with the confined nanogap geometry.

To further probe molecular ordering, two-dimensional grazing-incidence X-ray diffraction (2D-GIXD) measurements were performed on pristine and blended films (Figure 2f,g). The pristine DPPTT film exhibited well-defined (*h*00) lamellar peaks in the out-of-plane (*q*_*z*_) direction and a pronounced (010) π–π stacking peak in the in-plane (*q*_*xy*_) direction, indicating a highly ordered edge-on orientation.^36,37^ Upon blending with IEICO-4Cl, the (010) π–π stacking signal becomes more pronounced along *q*_*z*_, while lamellar ordering is reinforced along *q*_*xy*_, indicating a transition toward a more face-on molecular orientation (Figure 2f). Notably, the 1:0.25 blend exhibits enhanced face-on ordering without detectable IEICO-4Cl crystalline diffraction peaks. In contrast, distinct IEICO-4Cl diffraction features emerge in the 1:1 film (arrows in Figure 2g). This behavior correlates with the increased surface roughness observed in AFM. It indicates that excessive acceptor loading leads to crystalline phase segregation. These results suggest that the 1:0.25 composition forms a molecularly intermixed BHJ morphology that is well suited for the confined nanogap channel.

To directly probe nanoscale compositional distribution within BHJ nanograins, scattering-type scanning near-field optical microscopy (s-SNOM) combined with Fourier-transform infrared (FT-IR) nanospectroscopy was employed.^38^ Reference FT-IR spectra of pristine films exhibit characteristic vibrational signatures corresponding to the C–Cl stretching mode of IEICO-4Cl (1535 cm^–1^) and the C=O stretching mode of DPPTT (1664 cm^–1^) (Figure S13, Supporting Information). s-SNOM nanospectra (Figure 2h, left) reveal the simultaneous presence of both vibrational modes within individual nanograins, confirming nanoscale intermixed donor–acceptor domains. In contrast, the 1:1 film (Figure 2h, right) predominantly exhibits DPPTT-related features, indicative of donor-rich surface regions arising from phase segregation. These observations provide direct spectroscopic evidence that the 1:0.25 composition forms a molecularly homogeneous BHJ capable of supporting efficient photogeneration within the nanogap channel.

To examine how the blend composition affects trap-mediated charge accumulation within the nanogap channel, we further performed device-level capacitance–voltage (C–V) measurements on nanogap OPDs with different DPPTT:IEICO-4Cl blend ratios. As shown in Figure S14 (Supporting Information), the C–V curves measured at 100 Hz show clear composition-dependent capacitance responses. The pristine DPPTT device (1:0) shows a weak capacitance peak at a relatively high bias, indicating limited trap-mediated charge accumulation. Upon incorporation of IEICO-4Cl, the optimized DPPTT:IEICO-4Cl (1:0.25) blend shows the most pronounced and well-defined capacitance peak, which appears at the lowest bias among the investigated compositions. This lower peak voltage indicates that trap-mediated/interfacial charge accumulation is activated more readily in the optimized nanogap channel, while the enhanced peak intensity reflects more efficient charge storage associated with the photomultiplication process.^39,40^ In contrast, the 1:0.5 blend shows a capacitance peak shifted to a higher bias together with a reduced peak capacitance, while the 1:1 blend exhibits a substantially suppressed, broadened, and unstable capacitance response, suggesting less favorable charge accumulation and increased electrical disorder in the nanogap device. Therefore, these composition-dependent C–V responses demonstrate that although the active nanogap channel has an extremely small volume, its electrical charge-accumulation behavior is strongly governed by the BHJ blend composition. This result provides device-level electrical evidence linking the optimized BHJ composition to efficient trap-mediated charge accumulation and enhanced photomultiplication performance.

To understand the origin of the exceptionally high photocurrent generated within the deeply confined nanogap channel, we first examined whether plasmonic field enhancement occurs at the Au/Al junction. Figure 3a shows the electric field distribution simulated using a three-dimensional finite-difference time-domain (3D-FDTD) method. A strong localization of the optical field is observed exclusively under transverse-magnetic (TM)-polarized excitation, while negligible enhancement appears under transverse-electric (TE) polarization. This polarization selectivity is a characteristic signature of surface plasmon polariton (SPP) excitation, which occurs only when the incident field has an electric field component normal to the metal–dielectric interface.^41,42^ In this coplanar nanogap geometry, the closely spaced Au and Al edges strongly confine the TM-polarized electric field within the ∼10 nm channel, resulting in a highly localized gap-plasmon mode.^43–45^ Consistently, optical absorption cross-section analysis at 830 nm shows an effective absorption width comparable to the physical nanogap width, confirming that light absorption contributing to photocurrent generation is confined to the nanogap channel (Figure S15, Supporting Information). To quantify this effect, polarization-dependent optical simulations were performed (Figure 3b). Under TM-polarized illumination, absorption within the nanogap is significantly enhanced relative to the TE case across the simulated wavelength range.^46^ At the device operating wavelength of 830 nm, the simulated absorption within the nanogap under TM-polarized illumination is nearly 2 orders of magnitude higher than that under TE-polarized illumination, confirming that gap-plasmon excitation dramatically enhances local optical absorption in the nanoscale photoactive region.

**Figure 3:**
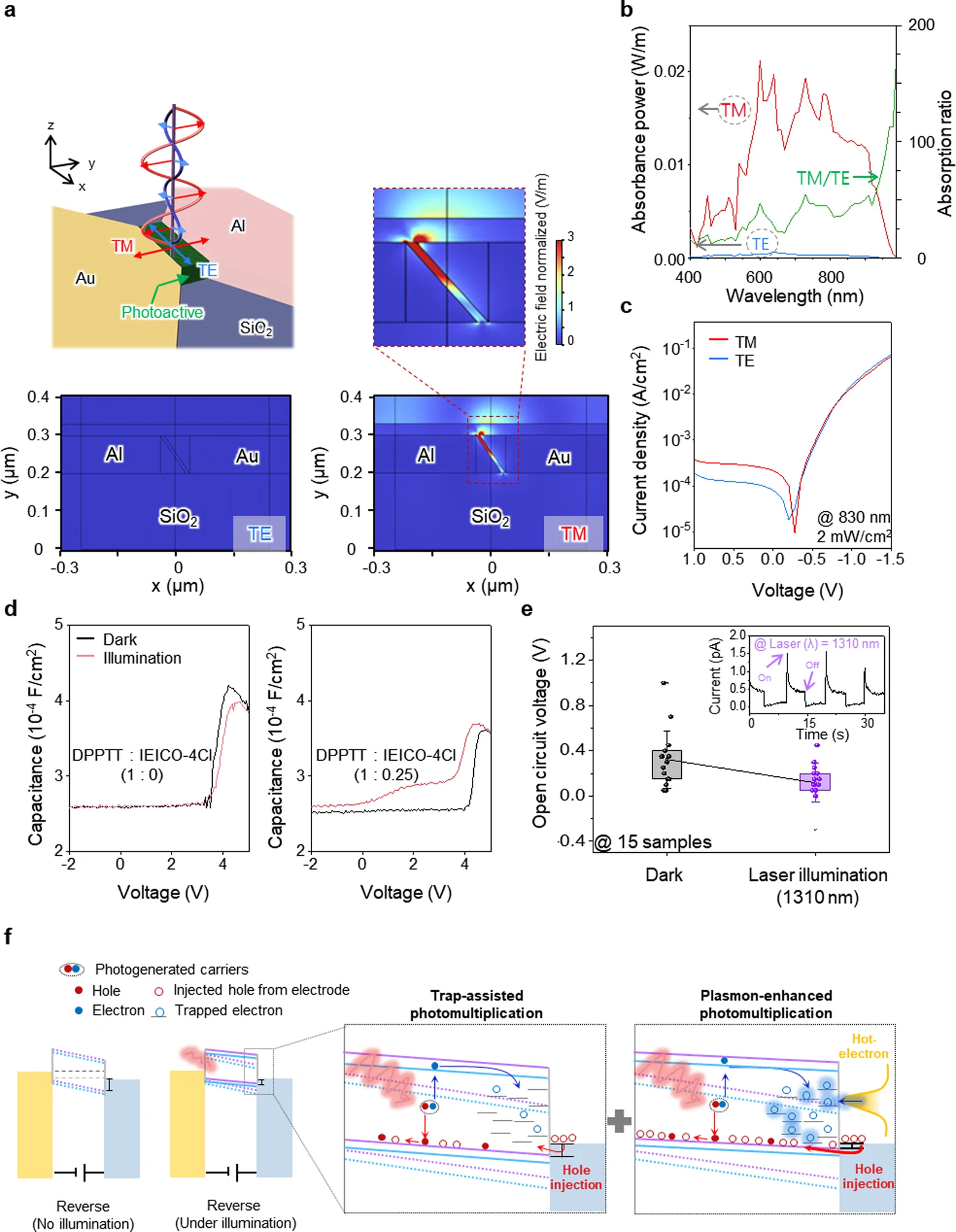
Plasmonic field enhancement and gain mechanism in PN-OPDs. (a) Schematic illustration of polarization-dependent excitation in the nanogap, where TM-polarized light can more effectively couple to confined plasmonic modes within the nanoscale gap compared to TE polarization. 3D-FDTD simulated electric-field distribution of PN-OPD under 830 nm TE- and TM-polarized illumination. (b) Simulated wavelength-dependent absorbance of the PN-OPD nanogap structure obtained from 3D-FDTD calculations. (c) I–V characteristics of the PN-OPD measured under 830 nm TM- and TE-polarized light. (d) Capacitance–voltage characteristics of the PN-OPD with D: A ratios of 1:0.25 and 1:1, measured under dark conditions and under 830 nm illumination. (e) Open-circuit voltage of 15 PN-OPD devices measured under dark and 1310 nm illuminated conditions. (Inset) Time-resolved photocurrent response of the PN-OPD under 1310 nm illumination measured at a bias of 1 V. (f) Energy band diagrams comparing (left) trap-assisted photomultiplication and (right) plasmon-enhanced photomultiplication, where plasmon-induced hot electrons accelerate trap-assisted charge accumulation, leading to enhanced band bending and hole injection.

We experimentally validate this polarization dependence by measuring the photocurrent under TE- and TM-polarized illumination at 830 nm (Figure 3c). The TM-polarized photocurrent is approximately 2.5 times larger than the TE-polarized response, consistent with the simulated field-enhancement behavior. Because the BHJ film itself is optically isotropic, this polarization anisotropy confirms that the enhanced photocurrent originates from plasmonic effects confined within the nanogap channels. The lower experimental TM/TE enhancement ratio compared with the simulated value may be explained by the fact that the TM response is more strongly degraded by nonidealities in the actual nanogap geometry, such as nanoscale gap-size nonuniformity, metal-edge roughness, and local structural imperfections.^47,48^ These results demonstrate that gap-plasmon excitation enhances absorption and carrier generation within the nanogap, enabling strong photocurrent generation despite the extremely small active area.

In addition to plasmonic field enhancement, the PN-OPD exhibits trap-assisted photomultiplication (PM) behavior, as evidenced by the donor-rich composition of the optimized DPPTT:IEICO-4Cl (1:0.25) blend and the sublinear photocurrent–power dependence (α ≈ 0.5; Figure 2c). This PM behavior originates from the limited acceptor content, which promotes electron trapping within the polymer matrix and at the Al/BHJ interface in the nanogap channel. To probe this trap-assisted process, we examine the capacitance–voltage (C–V) characteristics of the PN-OPD under illumination (Figure 3d). In pristine DPPTT devices, illumination reduces the capacitance, reflecting rapid recombination and negligible carrier accumulation. In contrast, the 1:0.25 BHJ nanogap device shows a pronounced increase in capacitance and a shift of the peak capacitance voltage toward lower bias under illumination. This shift reflects the accumulation of photogenerated electrons at the Al/BHJ interface, which induces upward band bending and reduces the effective barrier for hole injection from the Al electrode.^44–46^ Illumination also gives rise to a broad capacitance shoulder near ∼2 V that is absent in the dark. This feature indicates the presence of excess mobile charges that do not immediately recombine.^47–49^ Because electron accumulation lowers the injection barrier, these excess charges are attributed to photoinjected holes that remain stored near the interface, providing further evidence of illumination-induced charge accumulation (Figure 3f, left). These results provide direct evidence that trap-assisted photomultiplication occurs even within the confined BHJ nanogap channel, enabling high responsivity and exceptionally large quantum efficiencies.

Despite the trap-assisted nature of the PM process, the PN-OPD retains a relatively fast temporal response. In a simplified photoconductive gain picture, the PM gain can be expressed as^49^ $G\approx \frac{\tau_{\mathrm{life}}}{\tau_{\mathrm{tr}}}$ , where *τ*_life_ represents the effective lifetime associated with trapped photogenerated carrier and *τ*_tr_ is the transit time of the mobile carriers. Under a drift-dominated approximation, the transit time can be expressed as, $\tau_{\mathrm{tr}}\approx \frac{L}{\mu E}=\frac{L^{2}}{\mu V_{\mathrm{eff}}}$ where *L, μ, E*, and *V*_eff_ denote the carrier transport length, mobility, electric field, and applied voltage, respectively. This relationship indicates a strong dependence on the carrier transport length. Whereas the transport distance in conventional vertical PM-OPDs is typically determined by active-layer thicknesses on the order of 10^2^ nm, the transport distance in the present PN-OPD is defined by the ∼10 nm nanogap. This ultrashort transport distance can substantially reduce the carrier transit time and thereby provide a favorable condition for achieving appreciable photomultiplication without necessarily requiring an excessively long trapped-carrier lifetime. The nanogap geometry can therefore help mitigate the conventional gain–speed trade-off of trap-assisted PM, providing a plausible explanation for the relatively fast photoresponse observed in the PN-OPD. The measured temporal response may additionally be influenced by trapping/detrapping dynamics, interfacial carrier injection, and RC effects.

Plasmon-induced hot-carrier injection may further contribute to carrier generation and injection in the PN-OPD. When surface plasmon polaritons (SPPs) undergo nonradiative decay in the deeply confined nanogap, energetic (“hot”) carriers can be generated in the metal and subsequently injected across the metal–semiconductor interface if they possess sufficient energy and momentum to overcome the local interfacial barrier.^50,51^ Hot-carrier-mediated sub-bandgap photodetection through internal photoemission across metal–semiconductor Schottky barriers is a well-established mechanism, and such hot-carrier-mediated internal photoemission has also been demonstrated in organic semiconductor Schottky junctions.^52^

To examine whether such an interfacial process could contribute to the PN-OPD response, we investigated photocurrent generation under sub-bandgap excitation, where the BHJ absorption is negligible. As shown in Figure S16 (Supporting Information), Urbach analysis of the absorption edge shows only a limited band-tail contribution, and the extrapolated tail absorption at 1310 nm is negligible. Interestingly, under 1310 nm illumination—below the absorption edge of the DPPTT: IEICO-4Cl BHJ—the PN-OPD still exhibits measurable photocurrent transients and a noticeable shift in open-circuit voltage (Figure 3e). This result suggests that the 1310 nm photoresponse is unlikely to be predominantly governed by intrinsic sub-bandgap or band-tail absorption of the BHJ photoactive layer. Given the negligible BHJ absorption at 1310 nm, these observations are consistent with a contribution from plasmon-mediated hot-carrier generation at the metal nanogap, followed by carrier injection across the metal/BHJ interface. Within this interpretation, the injected carriers are preferentially trapped at the Al/BHJ interface, thereby increasing interfacial charge accumulation, lowering the hole-injection barrier, and inducing the observed Voc shift.^53,54^ Consistently, capacitance–voltage (C–V) measurements under 1310 nm illumination (Figure S17, Supporting Information) reveal an additional capacitance shoulder near 1.5 V, indicating the presence of excess injected carriers accumulated at the interface. This illumination-induced carrier injection and accumulation can further modulate the interfacial injection barrier, thereby contributing to the photomultiplication process (Figure 3f, right).

Furthermore, to examine a possible photothermal contribution to the 1310 nm photoresponse of the PN-OPD, we measured the device characteristics at an elevated temperature of 35 °C. As shown in Figure S18a (Supporting Information), thermal heating alone caused a slight increase in dark current together with a voltage shift, indicating that thermally assisted transport can contribute to the background current. Notably, the actual temperature rise induced by 1310 nm illumination under our measurement conditions is expected to be much smaller than this externally applied temperature increase because the DPPTT:IEICO-4Cl BHJ shows negligible absorption at 1310 nm and the underlying Si substrate can act as an efficient heat sink for heat generated in the metal electrodes. However, when the device, maintained at 35 °C, was additionally illuminated at 1310 nm, a distinct and reversible photocurrent response was observed (Figure S18b, Supporting Information). The transient photocurrent under 1310 nm illumination also showed fast and reproducible switching behavior without the slow baseline evolution typically expected for device-level photothermal heating and cooling. These observations suggest that thermally assisted transport may contribute to the background current, but it is insufficient to account for the distinct and fast light-induced photocurrent observed under 1310 nm illumination. Although these measurements do not directly resolve the generation and interfacial transfer of nonequilibrium hot carriers, the overall experimental results are consistent with metal/BHJ interfacial hot-carrier injection as a physically plausible contribution to the 1310 nm photoresponse. Further spectroscopic investigations, such as ultrafast transient absorption or time-resolved photoemission spectroscopy,^55,56^ would be valuable for directly elucidating plasmon-mediated hot-carrier injection in the PN-OPD.

Figure 4a shows optical microscopy (OM) images of the PN-OPD integrated within a polymer waveguide-based photonic circuit with a 1 × 2 multimode interference (MMI) beam splitter, which can divide the optical power in half. The MMI enables controlled spatial redistribution of the guided optical field through self-imaging, generating well-defined interference patterns at the output waveguides. By positioning the PN-OPD at the MMI output, the device directly converts routed optical signals into electrical current, enabling evaluation of both waveguide-mediated light propagation and detector response within an integrated photonic architecture. Scanning electron microscopy (SEM) images provide magnified views of the MMI region and the integrated nanogap photodetector with simple tapered structures. The SU-8 waveguide is patterned directly above the nanogap channel, enabling evanescent-field coupling between the guided optical mode and the underlying photoactive region at 852 nm. The fabrication procedure is described in Methods and Figure S1 (Supporting Information). To ensure that light absorption occurs only at the nanogap detector region, the photoactive layer outside the detection area is selectively removed using a two-step SU-8 patterning process. First, a thin SU-8 layer is patterned to locally encapsulate the nanogap channel, serving as a protective mask during subsequent O_2_ plasma etching. The exposed photoactive material outside the channel region is then removed, effectively suppressing parasitic absorption in the waveguide. Subsequently, an additional thick SU-8 layer is patterned to define the waveguide structure directly above the encapsulated channel. The resulting waveguide retains the designed rectangular geometry with well-defined dimensions after the two-step SU-8 patterning (Figure S19, Supporting Information). The I–V curves measured after waveguide deposition show negligible changes (Figure S20, Supporting Information), indicating that waveguide integration process does not significantly alter the electrical transport characteristics of the PN-OPD. The stability of the waveguide-integrated PN-OPD was further evaluated under both ambient storage and continuous operation conditions. After storage under ambient conditions (25 °C, 50% RH) for 12 days, the dark current and photoresponse remained nearly unchanged compared with the initial device characteristics (Figure S21, Supporting Information). In addition, under repeated 1 Hz light on/off modulation for 1 h, the device exhibited highly reproducible photocurrent switching after a brief initial stabilization period, with no noticeable degradation in photocurrent amplitude or switching characteristics (Figure S22, Supporting Information). The good stability is likely attributable in part to the SU-8 waveguide layer, which also serves as a protective encapsulation layer for the nanogap channel.

**Figure 4:**
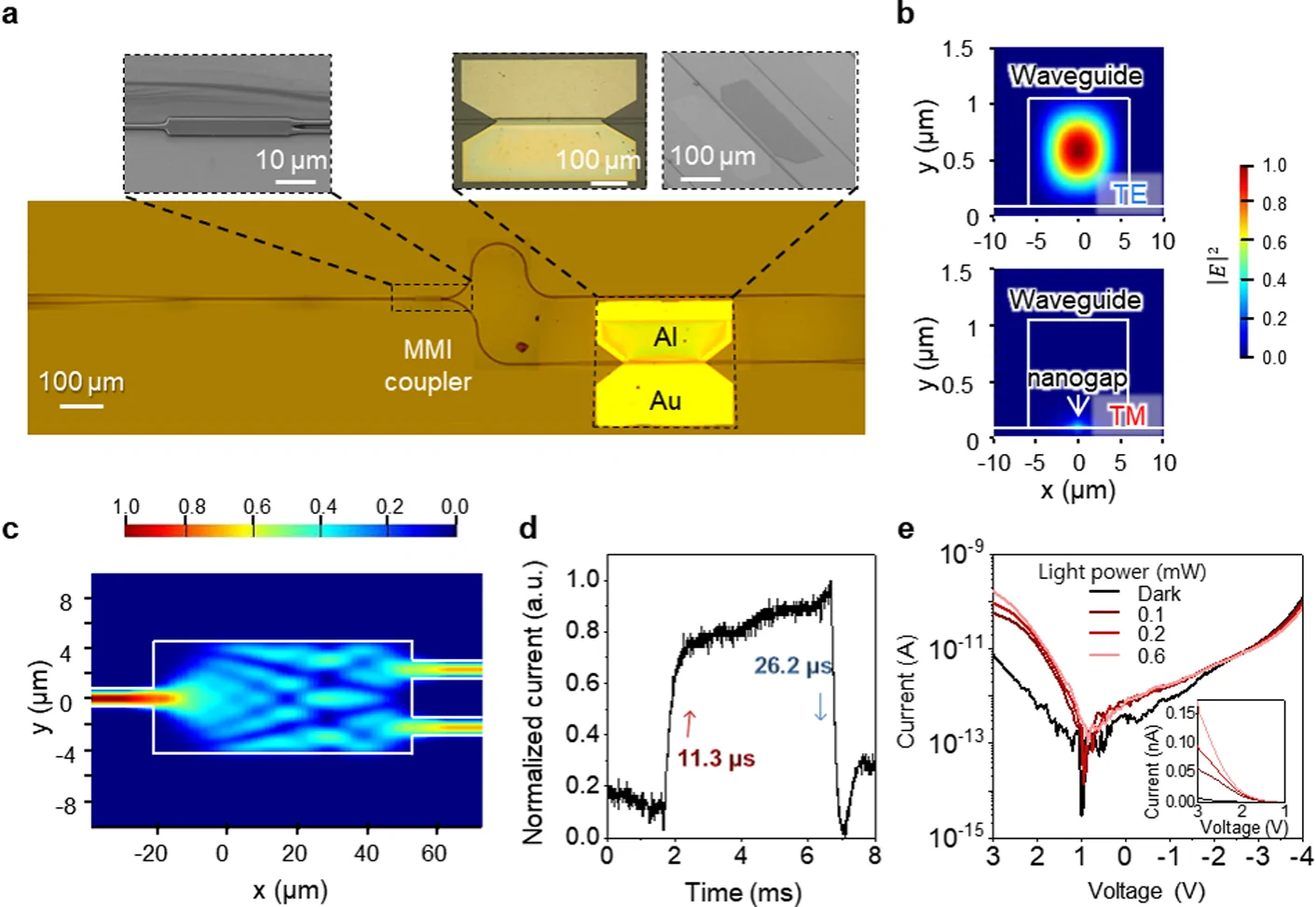
Demonstration of waveguide-integrated photodetection in PN-OPDs. (a) (Top) OM image of the polymer waveguide-integrated PN-OPD, showing the optical path from the input waveguide through the MMI region to the detector. (Bottom) Corresponding schematic illustration of the waveguide-integrated device architecture, showing the full optical routing path from the input waveguide through the MMI region to the PN-OPD. Enlarged views of the MMI and PN-OPD regions are highlighted by blue and red boxes, respectively, where the MMI is shown by SEM and the PN-OPD by OM. (b) Simulated electric-field distribution in the PN-OPD for TE- and TM-polarized guided modes at 852 nm, showing polarization-dependent coupling into the nanogap channel, with enhanced field localization under TM polarization. (c) Simulated electric-field intensity distribution in the MMI waveguide (*W* = 9 μm, *L* = 73 μm) at 852 nm, showing self-imaging behavior along the propagation direction. (d) Transient photocurrent response of the PN-OPD under guided light, showing on/off switching. (e) Current–voltage (I–V) characteristics of the PN-OPD measured under guided light from the MMI output with varying light intensities, demonstrating that the device responsively tracks the modulated optical signal. The inset shows a magnified view of the reverse-bias region plotted on a linear scale.

A comparison with representative waveguide-integrated photodetectors is provided in Table S2 (Supporting Information). Although the bandwidth of the PN-OPD is lower than those of GHz-class inorganic and 2D-material-based waveguide photodetectors, it exhibits a high responsivity, together with an ultralow dark current. Notably, previously reported waveguide-integrated organic photodetectors were limited to visible-wavelength operation and generally lacked comprehensive performance metrics. These results highlight the potential of the plasmonic nanogap architecture for high-gain organic photodetection in integrated photonic circuits.

To elucidate the coupling of guided light into the nanogap channel, electromagnetic simulations were performed using the extracted waveguide geometry (width of 12 μm and thickness of 1.2 μm) based on a finite-difference eigenmode (FDE) method (Figure 4b). At the operating wavelength of 852 nm, the transverse electric (TE) mode remains largely confined within the waveguide core, exhibiting minimal interaction with the nanogap region. In contrast, the transverse magnetic (TM) mode shows significant electric-field leakage into the nanogap, exciting a gap-plasmon mode and enabling efficient plasmon-assisted evanescent coupling. In addition, the optical field distribution within the MMI region is simulated to evaluate waveguide-based signal routing (Figure 4c). The MMI structure exhibits clear self-imaging behavior along the propagation direction, resulting in equal power splitting into two output waveguides at a device length of 73 μm. This 50:50 splitting confirms that the MMI effectively redistributes the guided optical signal before photodetection, enabling controlled delivery of optical power to the PN-OPD at the output ports (Figure S23, Supporting Information). Experimentally, the optical power measured at the two output ports of the fabricated 1 × 2 MMI exhibited a splitting ratio of 51.3:48.7, which closely matches the simulated and designed 50:50 power distribution.

This coupling behavior is experimentally verified by launching 852 nm light into the waveguide and measuring the photocurrent response (Figure 4d,e). The photocurrent measured at the single-port of the 1 × 2 MMI output scales with the input optical power while closely following its modulation, confirming reliable optical-to-electrical signal conversion through the integrated waveguide. The transient response exhibits a rise time of 11.3 μs and a fall time of 26.2 μs, corresponding to a sub-MHz response speed consistent with the frequency-domain bandwidth. This response speed is sufficient for a range of NIR PIC applications, including on-chip optical sensing, power monitoring,^57^ resonator stabilization,^58^ and feedback control,^59^ which typically operate in the sub-MHz regime.

## Conclusion

In summary, we present a plasmonic nanogap organic photodetector that enables efficient photodetection within an extremely confined nanoscale channel while remaining directly compatible with planar photonic platforms. The nanogap architecture enhances light–matter interaction and carrier extraction within a deeply confined region, enabling high responsivity and quantum efficiency while maintaining low dark current. The combined experimental and simulation results reveal that the device performance arises from a synergistic interplay of plasmonic field confinement, hot-carrier injection, and trap-assisted photomultiplication. This multimechanism operation enables substantial photogain while preserving a relatively fast temporal response that significantly exceeds that of typical photomultiplication-based OPDs. These findings highlight that nanoscale plasmonic confinement can mitigate the conventional trade-off between gain and speed in organic photodetectors. Importantly, the coplanar nanogap geometry provides a natural interface with integrated photonic architectures. By enabling evanescent coupling with guided optical modes, the PN-OPD directly converts routed optical signals into electrical outputs, demonstrating its applicability in waveguide-integrated photonic circuits. This capability represents a key step toward bridging organic optoelectronic devices with on-chip photonic systems. More broadly, the combination of plasmonic nanogap confinement, photogain mechanisms, and waveguide compatibility provides a viable pathway toward scalable, functionally integrated organic photonic systems, thereby expanding the role of organic semiconductors in on-chip photonics.

## Methods

### Materials and Device Fabrication

DPPTT, poly­(3,6-bis­(5-bromo-thiophen-2-yl)-N, N-dialkyl-1,4-dioxo-pyrrole­[3,4-*c*]­pyrrole), and IEICO-4Cl (Ossila); 2,2′-((2Z,2′Z)-(((4,4,9-tris­(4-hexylphenyl)-9-(4-pentylphenyl)-4,9-dihydro-s-indaceno­[1,2-b:5,6-b:dithiophene-2,7-diyl)­bis­(4-((2-ethylhexyl)­oxy)­thiophene-5,2-diyl))­bis­(methanylylidene))­bis­(5,6-dichloro-3-oxo-2,3-dihydro-1H-indene-2,1-diylidene))­dimalononitrile, were used as the donor and acceptor materials, respectively. The bulk heterojunction (BHJ) solution was prepared by dissolving the materials in chlorobenzene with 1 wt % chloronaphthalene at donor-to-acceptor weight ratios of 1:*x* (*x* = 0.1, 0.25, 0.5, 1). The concentration of DPPTT was fixed at 10 mg mL^–1^. The solution was stirred overnight at room temperature and spin-coated onto the nanogap coplanar electrodes at 2500 rpm for 60 s, yielding a film thickness of ∼30 nm. The nanogap coplanar electrodes were fabricated via lithography,^25^ as shown in Supplementary Figure S1. Polymer waveguides with the self-aligned organic detector structure were fabricated by the two-step tapered waveguide structures with SU-8 photoresists. The first tapered waveguide structures with 500 nm-thick SU-8 2000.5 photoresist were formed to isolate the PN-OPD near and on the nanogap detector region. Then, O_2_ plasma was irradiated to remove the unnecessary absorber on the substrate except under the thin SU-8 taper. The second SU-8 waveguides, using SU-8 6001 structures, were then spin-coated at 5000 rpm for 60 s to obtain a ∼1.2 μm-thick film, which was patterned again by photolithography to define the main polymer waveguides with 1 × 2 MMI structures. Before coating the second SU-8 layer, the substrate was treated with hexamethyldisilazane (HMDS) to improve adhesion.

### Optical and Structural Characterization

Optical absorption spectra were measured using a UV–Vis–NIR spectrometer (Lambda 750, PerkinElmer), and film thicknesses were determined using a surface profiler (AlphaStep AS-IQ, KLA-Tencor). The nanogap structure and BHJ infiltration were characterized using transmission electron microscopy (Titan 80–300, FEI) and by electron-beam–induced current (EBIC) imaging in a scanning electron microscope (Inspect F50, Thermo Fisher Scientific), and the SU-8 waveguide thickness was determined by SEM. The morphologies of BHJ films were examined by optical microscopy (LV150N, Nikon) and atomic force microscopy (XE-100, Park Systems). Vibrational near-field imaging was performed using an s-SNOM setup coupled to a QCL source (MIRcat-QT-Z-2400, Daylight Solutions), following procedures reported previously.^38^ Two-dimensional grazing-incidence X-ray diffraction (2D-GIXD) measurements were conducted at the 3C-SAXS beamline of the Pohang Accelerator Laboratory.

### Electrical and Optoelectronic Characterization

Static current–voltage behavior under dark and illuminated conditions was measured using a semiconductor parameter analyzer (Keithley 4156B). Noise power spectral densities for D* extraction were obtained using an SR570 low-noise current preamplifier and an Advantest R9211B spectrum analyzer. Near-infrared and visible illumination was provided by 830- and 635-nm laser diodes (Thorlabs). Dynamic photoresponse measurements, including bandwidth determination, were carried out using a high-speed oscilloscope (Tektronix TDS2024) synchronized with a function generator (Tektronix AFG31000).

### Optical Simulations

Optical modes in the SU-8 waveguides were calculated by a finite-difference eigenmode (FDE) solver using Ansys Lumerical MODE. S-parameters, transmittances, and mode profiles in the 1 × 2 multimode interference (MMI) waveguides were also calculated by the eigenmode expansion (EME) solver using Ansys Lumerical MODE. The electric field distribution in the plasmonic nanogap structure was calculated by using the finite element method implemented in the RF module of COMSOL Multiphysics. The absorption in the nanogap region was calculated by integrating the time-averaged power dissipation density over the nanogap area, yielding the absorbed power per unit length in the out-of-plane direction. The complex refractive index of BHJ, determined by ellipsometry, was used in the simulations, while those of gold and aluminum were taken from previous reports.^60^

### Equations for Extracting Photodiode Parameters

*R* was obtained from

$$R\left[A/W\right]=\frac{\left(I_{\mathrm{light}}-I_{\mathrm{dark}}\right)}{P_{\mathrm{in}}A}$$

where *I*_light_ and *I*_dark_ are the currents under light illumination and in the dark, respectively; *P*_in_ is the light intensity measured by a power meter; and *A* is the channel area.

*EQE* was calculated as

$$\mathrm{EQE}\left[\%\right]=\left(\frac{Rhc}{q\lambda }\right)\times 100$$

where *h* is Planck̀s constant, *c* is the speed of light, *q* is the elementary charge, and λ is the wavelength of the incident light.

D* was calculated from

$$D^{*}\left[\mathrm{jones}\right]=\frac{R\sqrt{A}}{S_{n}}$$

where *S*_*n*_ is the noise current spectral density.

## Supplementary Material



## Notes

Additional details on device fabrication and waveguide integration; structural and morphological characterization of the PN-OPD and BHJ films; temperature-dependent dark-current analysis; control experiments on BHJ composition, thickness, and residual active-layer width; light-intensity-dependent photoresponse, EQE, transient response, and noise characteristics; capacitance–voltage and sub-bandgap photoresponse analyses; performance comparison with representative NIR photomultiplication-type OPDs; and optical simulations and characterization of the polymer waveguide and MMI structures (PDF)
