# Supplementary material for: Near-Infrared Plasmonic Nanogap Organic Photodetectors for On-Chip Photonic Integration
Source: ACS Nano. 2026 Sep 15;20(38):26062–73. doi: 10.1021/acsnano.6c08504 (PMC13629679; doi:10.1021/acsnano.6c08504)
Supplement: Supplementary file 1 [file nn6c08504_si_001.pdf]

Supporting information

Near-infrared Plasmonic Nanogap Organic Photodetectors for On-chip Photonic Integration

Danbi Kim[1,2], Hanna Lee[1,7], Changsoon Choi[3], Jung Pyo Hong[4,5], Do Kyung Hwang[4, 5, 8], Wassie M. Takele[6], Terefe G. Habteyes[6], Jeong Ho Cho[2], In-Ho Lee[4], Jae-Hoon Han[4*], and Jung Ah Lim[1, 7, 8, 9*]*

1. *Electronic and Hybrid Materials Research Center, Korea Institute of Science and Technology, Seoul 02792, Republic of Korea*
2. *Department of Chemical and Biomolecular Engineering, Yonsei University, Seoul 03722, Republic of Korea*
3. *School of Electronic and Electrical Engineering, Sungkyunkwan University, Suwon 16419, Republic of Korea*
4. *Center for Quantum Technology, Korea Institute of Science and Technology, Seoul 02792, Republic of Korea*
5. *KU-KIST Graduate School of Converging Science and Technology, Korea University, Seoul 02841, Republic of Korea*
6. *Department of Chemistry and Chemical Biology, University of New Mexico, Albuquerque, NM 87131, USA*

7. GIST InnoCORE AI-Nano Convergence Institute for Early Detection of Neurodegenerative Diseases, Gwangju Institute of Science and Technology, Gwangju 61005, Republic of Korea

8. Division of Nano and Information Technology, KIST School, University of Science and Technology (UST), Seoul 02792, Republic of Korea

9. Department of Materials Science and Engineering, YU-KIST Institute, Yonsei University, Seoul 03722, Republic of Korea

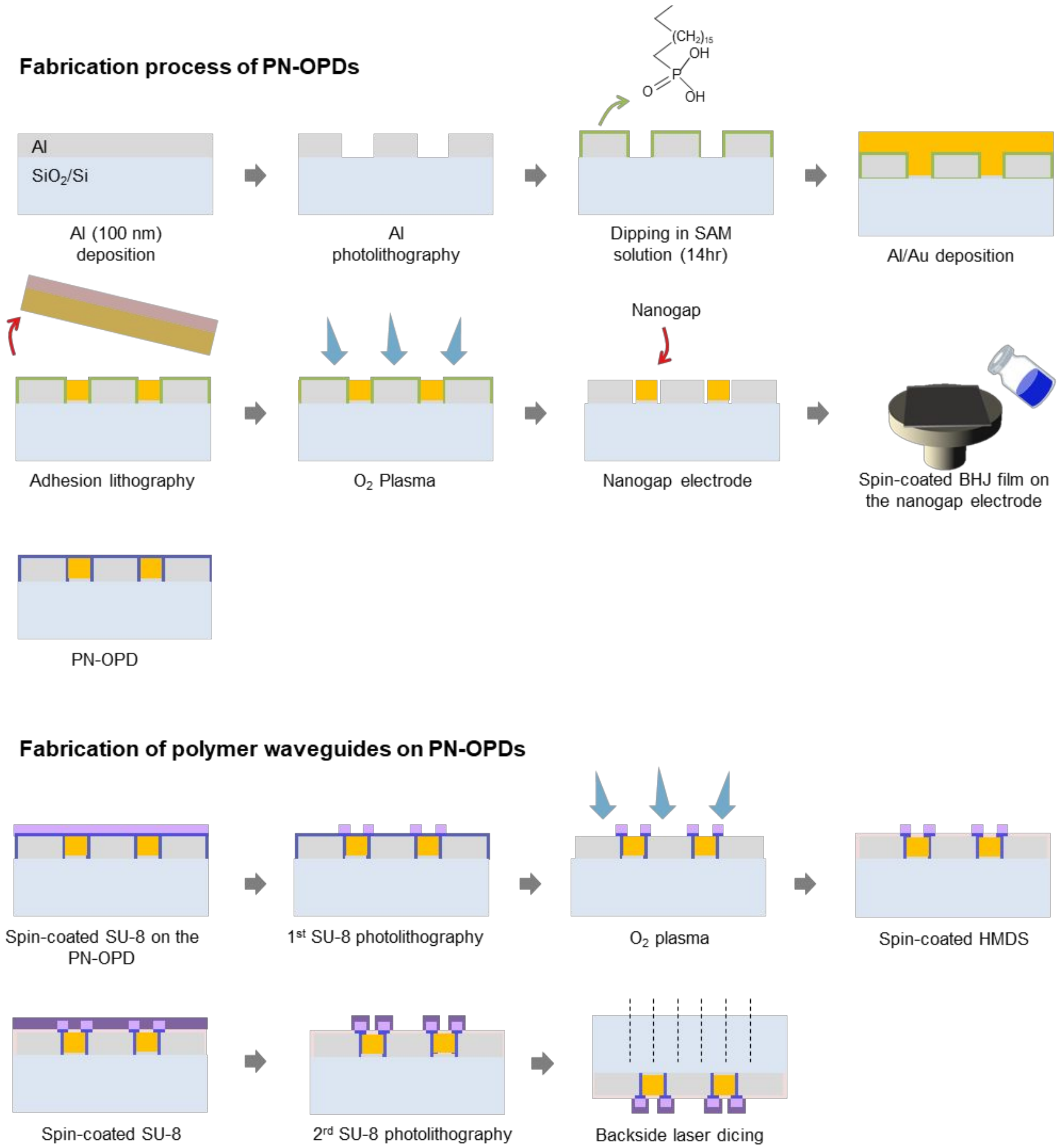

Figure S1. Fabrication of polymer waveguide-integrated PN-OPDs via adhesion photolithography.

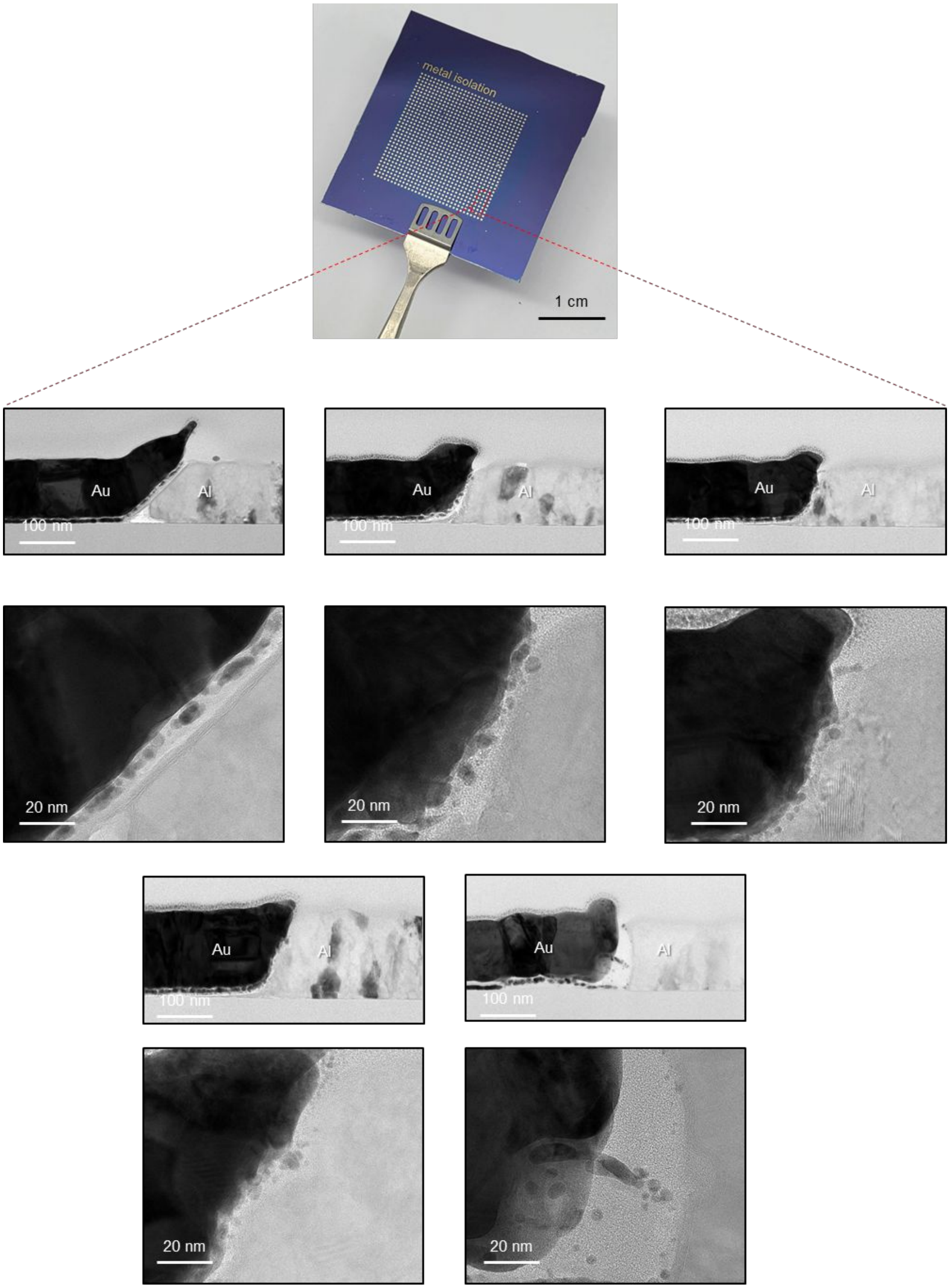

Figure S2. Optical image and cross-sectional TEM images of five different PN-OPD electrodes.

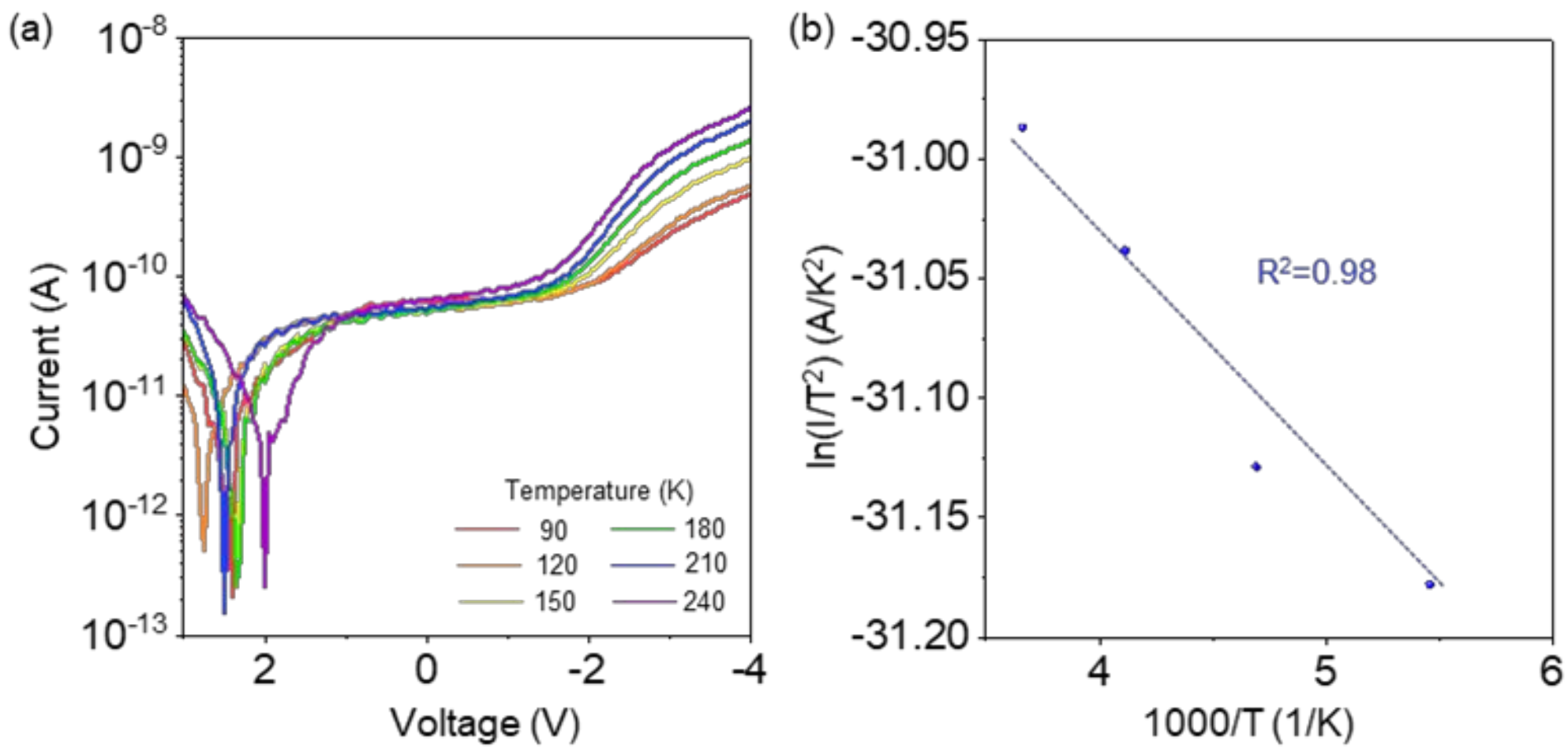

Figure S3. Temperature-dependent dark current analysis of the PN-OPD. (a) Temperature-dependent dark I–V characteristics measured over the temperature range of 90–240 K. (b) Arrhenius plot of the forward dark current measured at −4 V.

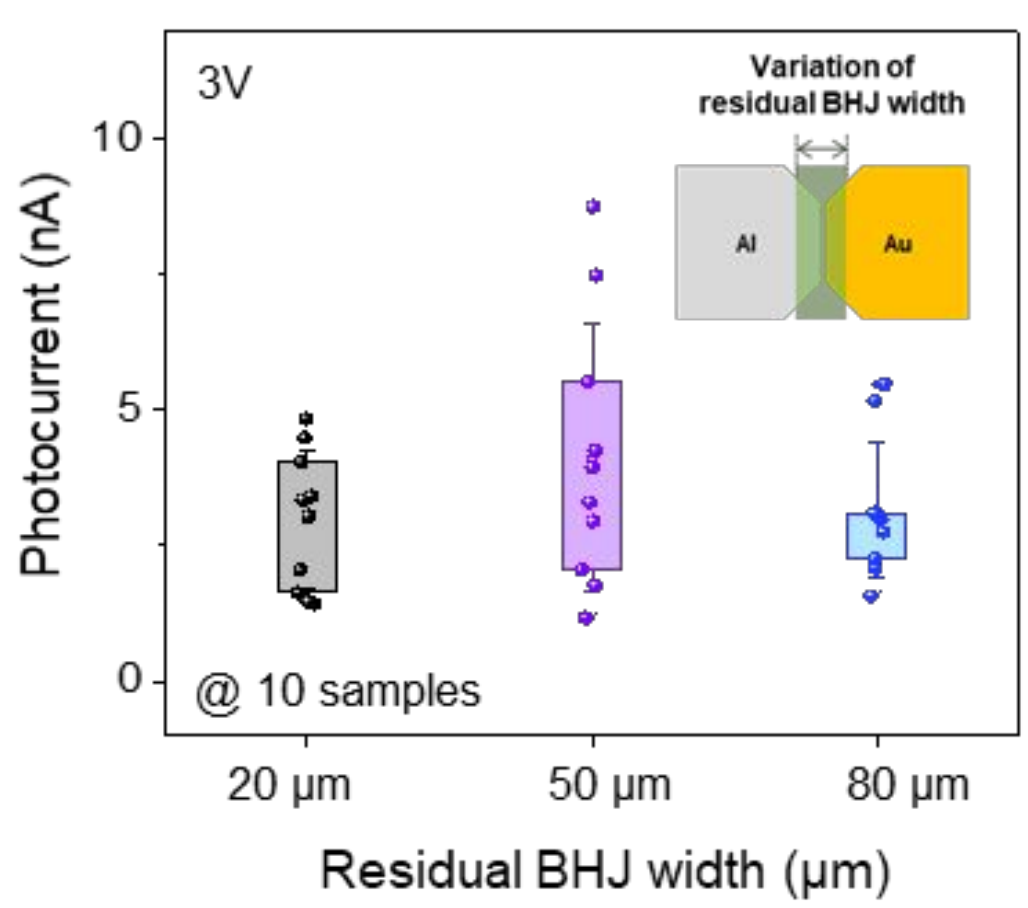

Figure S4. Photocurrent distributions of nanogap OPDs with different residual BHJ widths on the electrode regions. The photocurrent was measured at an applied bias of 3 V under 830 nm

illumination with a light intensity of 1 mW cm^{-2}. Each box plot represents data from 10 independent devices.

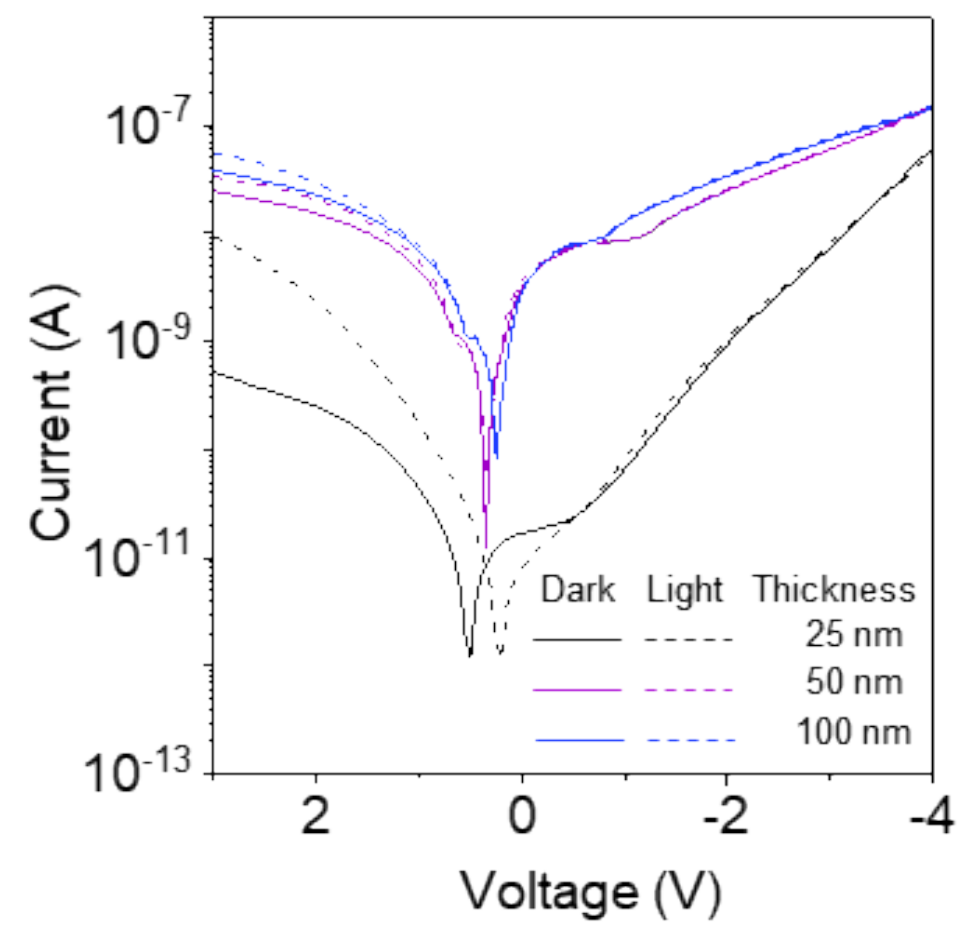

Figure S5. I-V characteristics of nanogap OPDs with different BHJ thicknesses of 25, 50, and 100 nm.

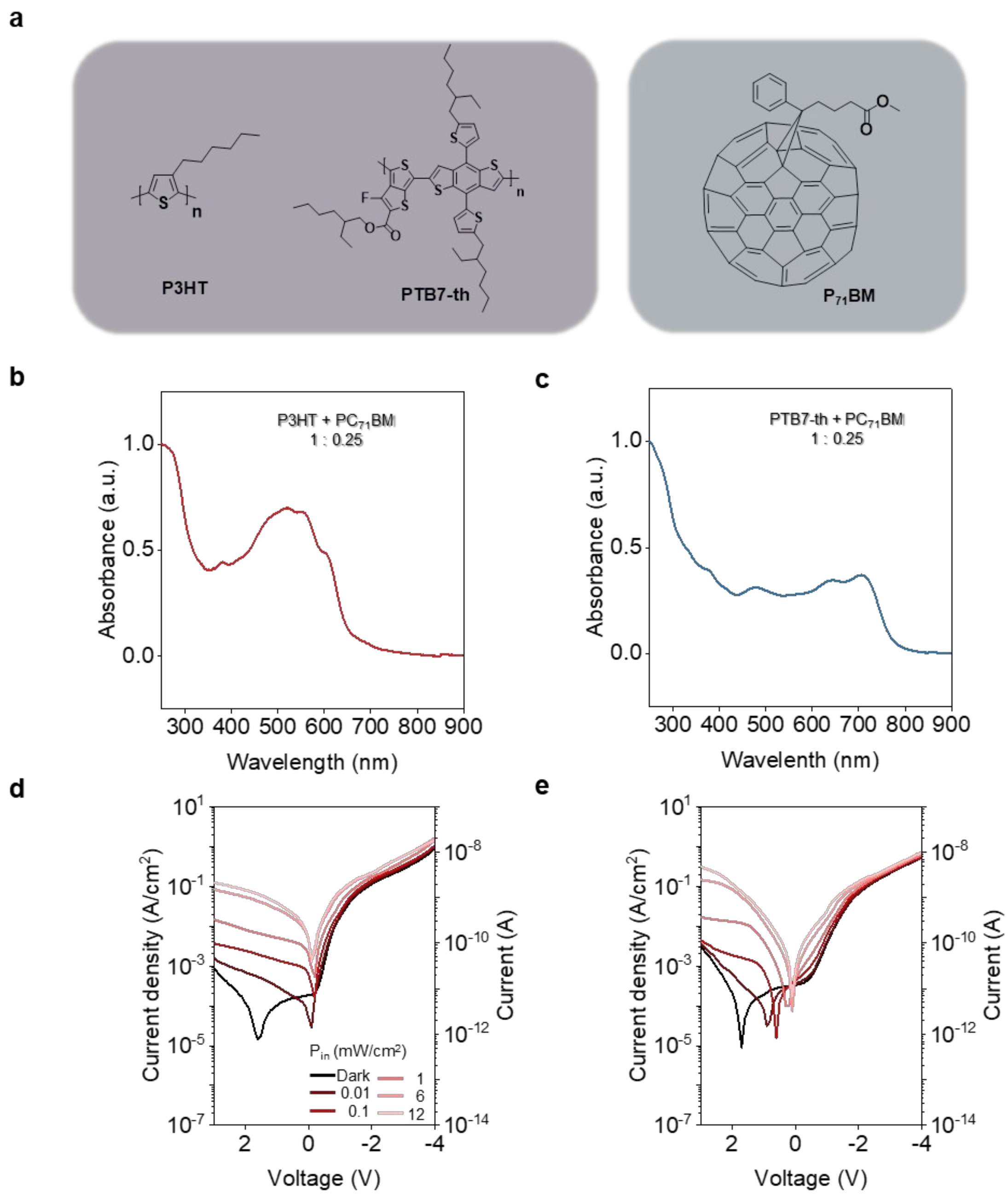

Figure S6. Chemical structures of the donor and acceptor materials used in this study. Optical absorption spectra of the BHJ films based on P3HT: $PC_{71}BM$ (b) and PTB7:$PC_{71}BM$ (c), showing their spectral responses. Current–voltage characteristics of PN-OPDs incorporating P3HT: PC_{71} BM (d) and PTB7:$PC_{71}BM$ (e) measured under varying light intensities, demonstrating the general applicability of the nanogap across various organic materials.

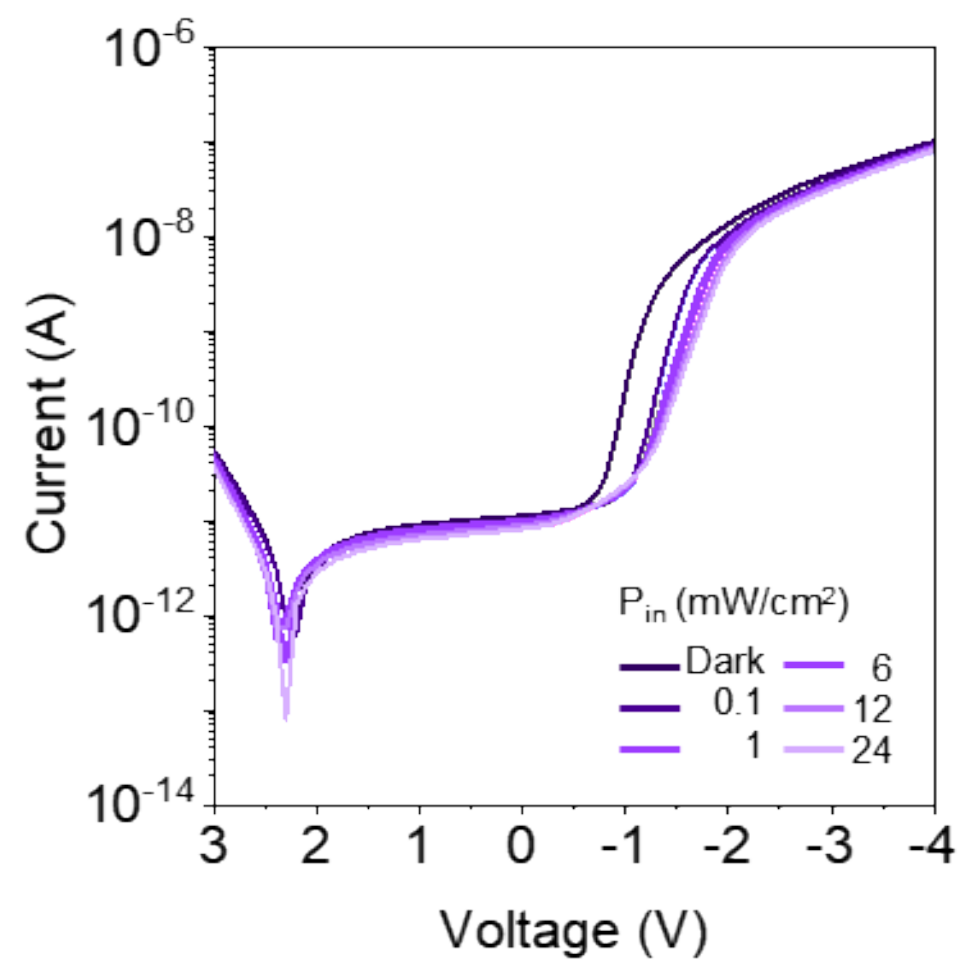

Figure S7. Photoresponse of PN-OPDs based on pristine DPPTT under 830 nm illumination according to light intensity.

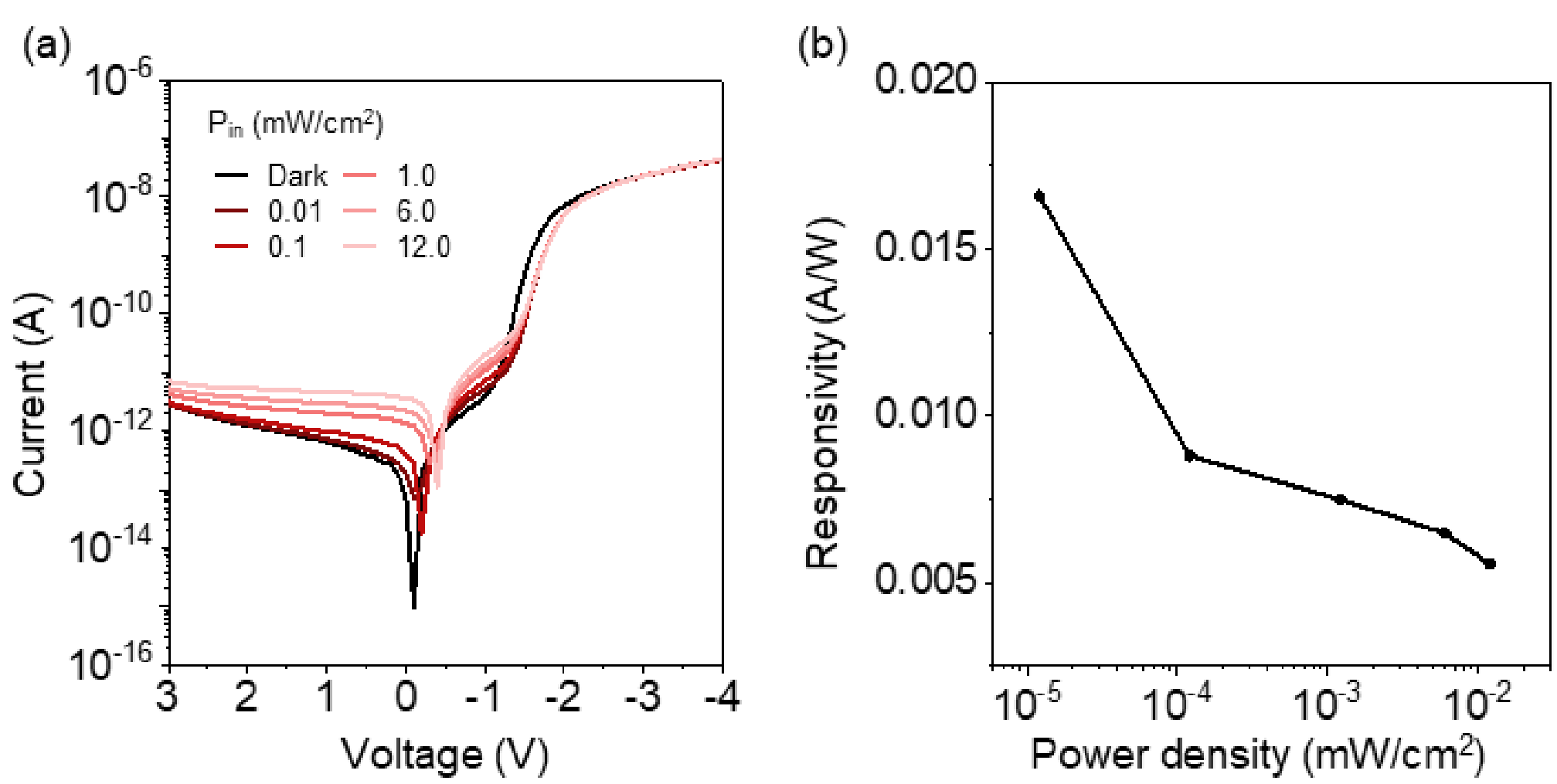

Figure S8. (a) Dark and illuminated current–voltage (I–V) characteristics of the PN-OPD with a donor: acceptor blend ratio of 1:0.1. (b) R of the 1:0.1 PN-OPD extracted at a bias of 1 V under varying incident power densities.

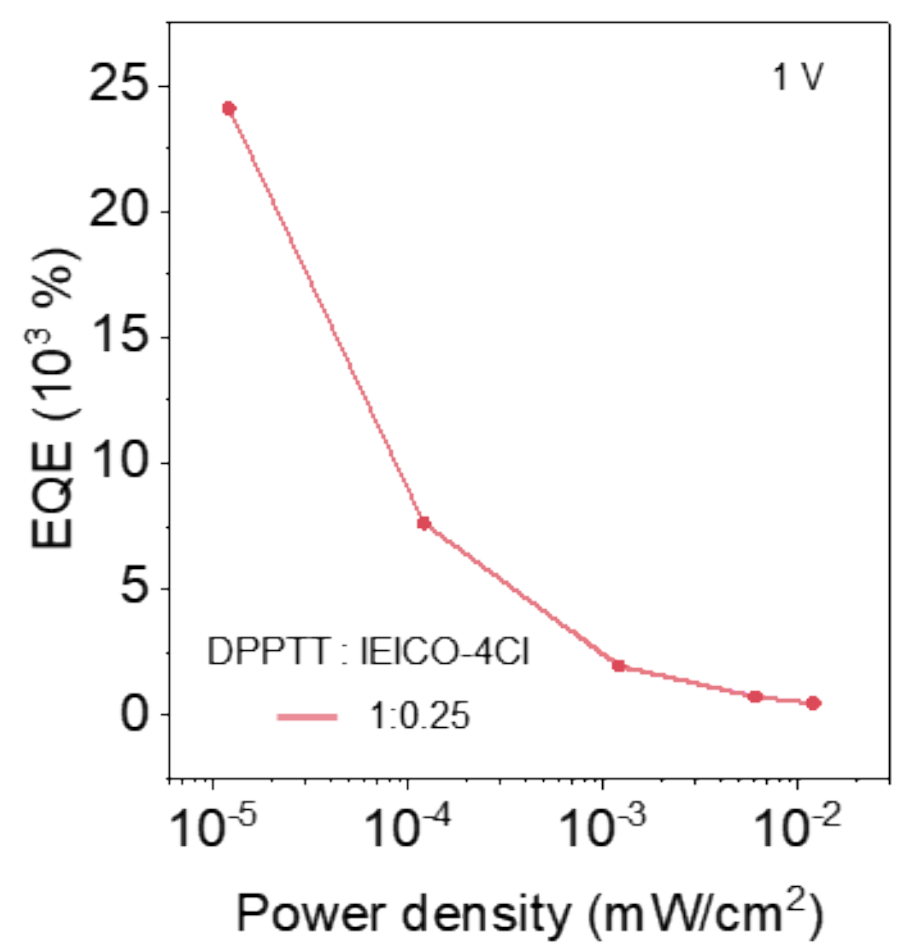

Figure S9. EQE of the 1:0.25 PN-OPD extracted at a bias of 1 V under varying incident power densities.

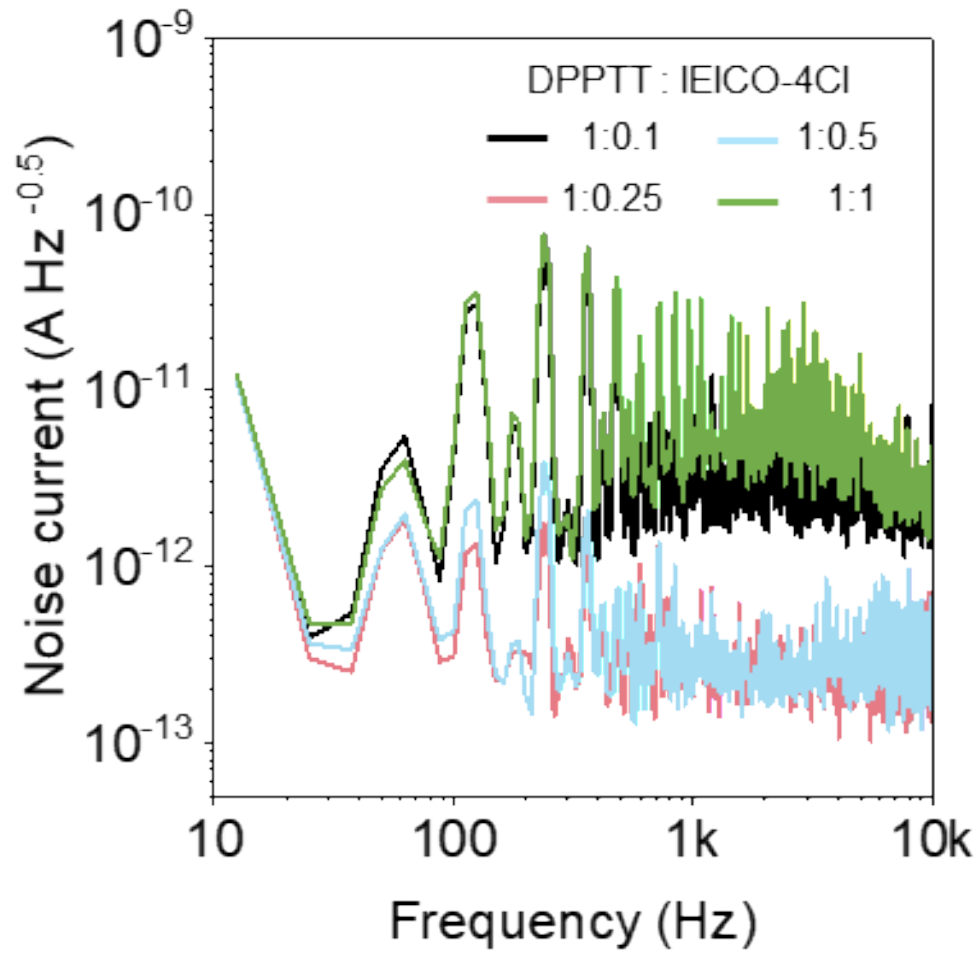

Figure S10. Noise current of PN-OPDs with various D: A ratios measured under dark conditions at an applied bias of 1 V.

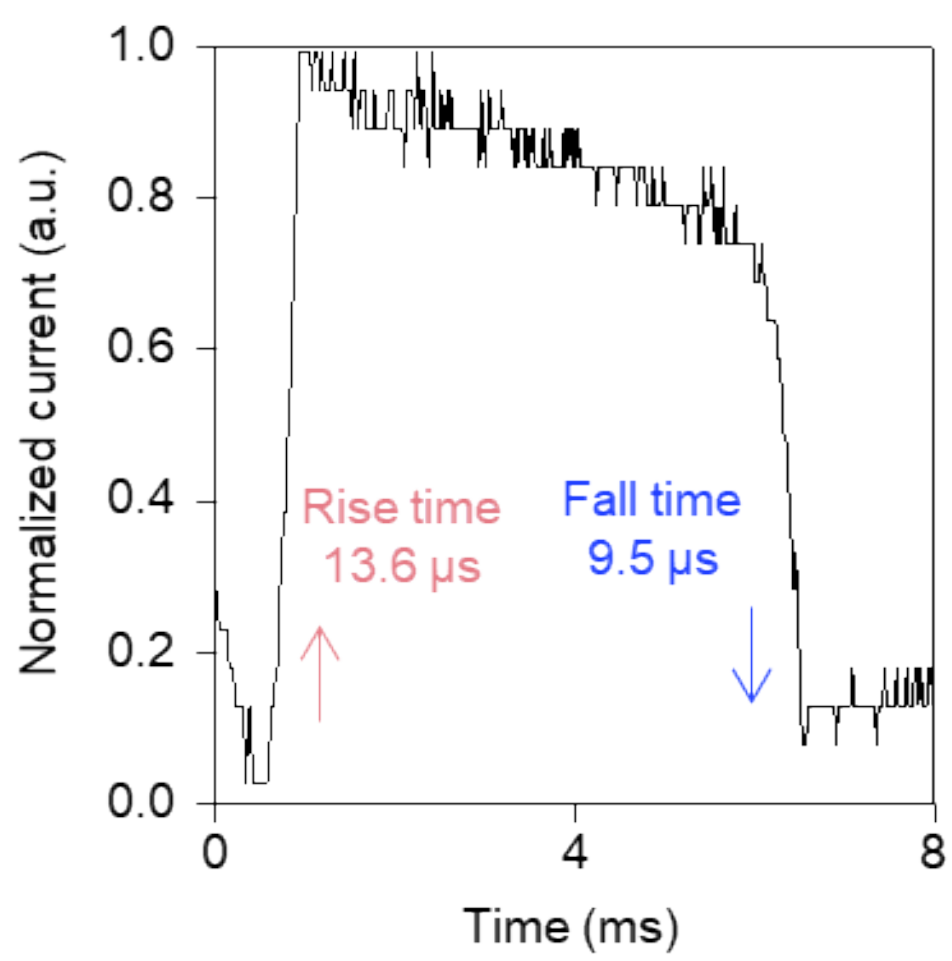

Figure S11. Transient photoresponse of the PN-OPD under free-space illumination.

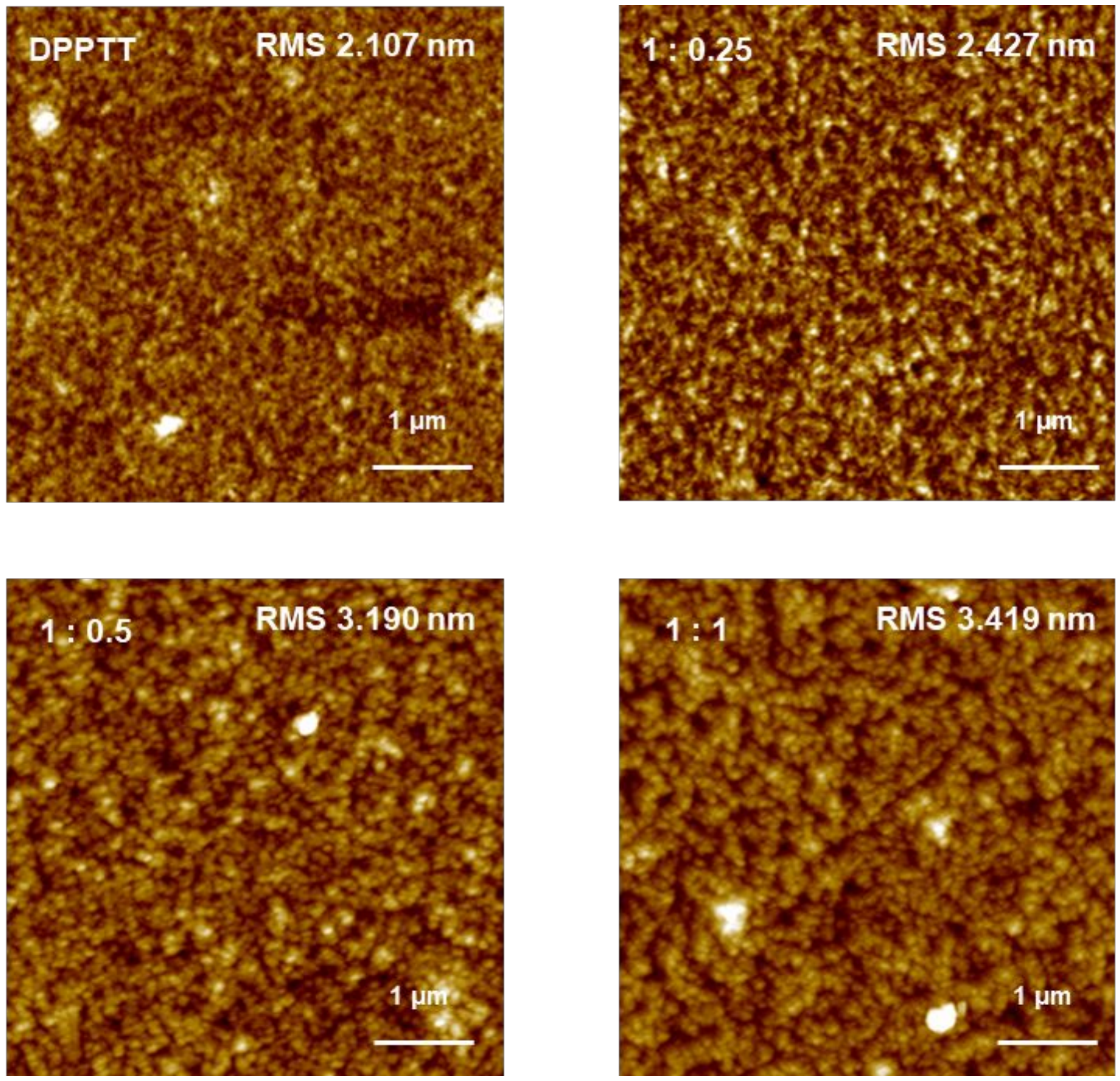

Figure S12. AFM height images of BHJ films with different DPPTT: IEICO-4Cl ratios.

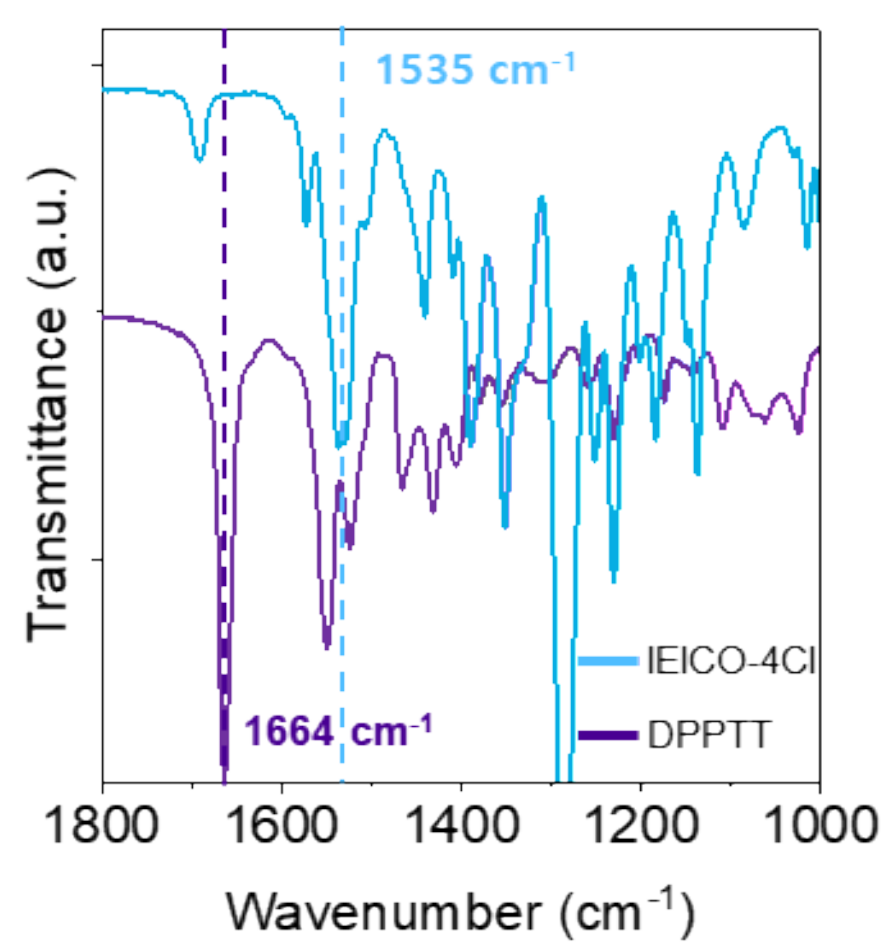

Figure S13. FT-IR spectra of pristine IEICO-4Cl and DPPTT films.

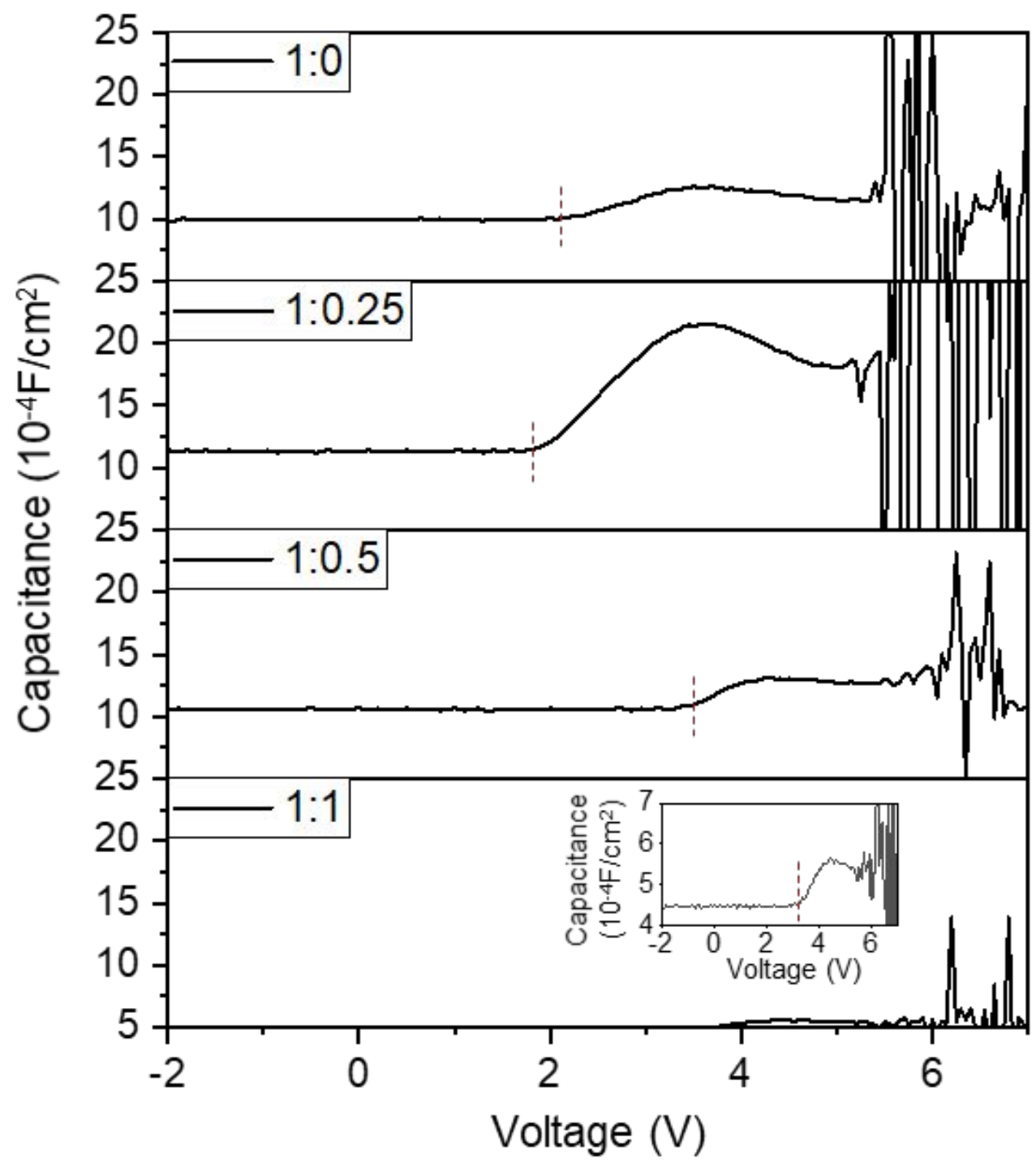

Figure S14. Capacitance–voltage (C–V) characteristics of the PN-OPD based on DPPTT: IEICO-4Cl BHJ blends with different blend ratios. The measurements were performed at 100 Hz in the dark.

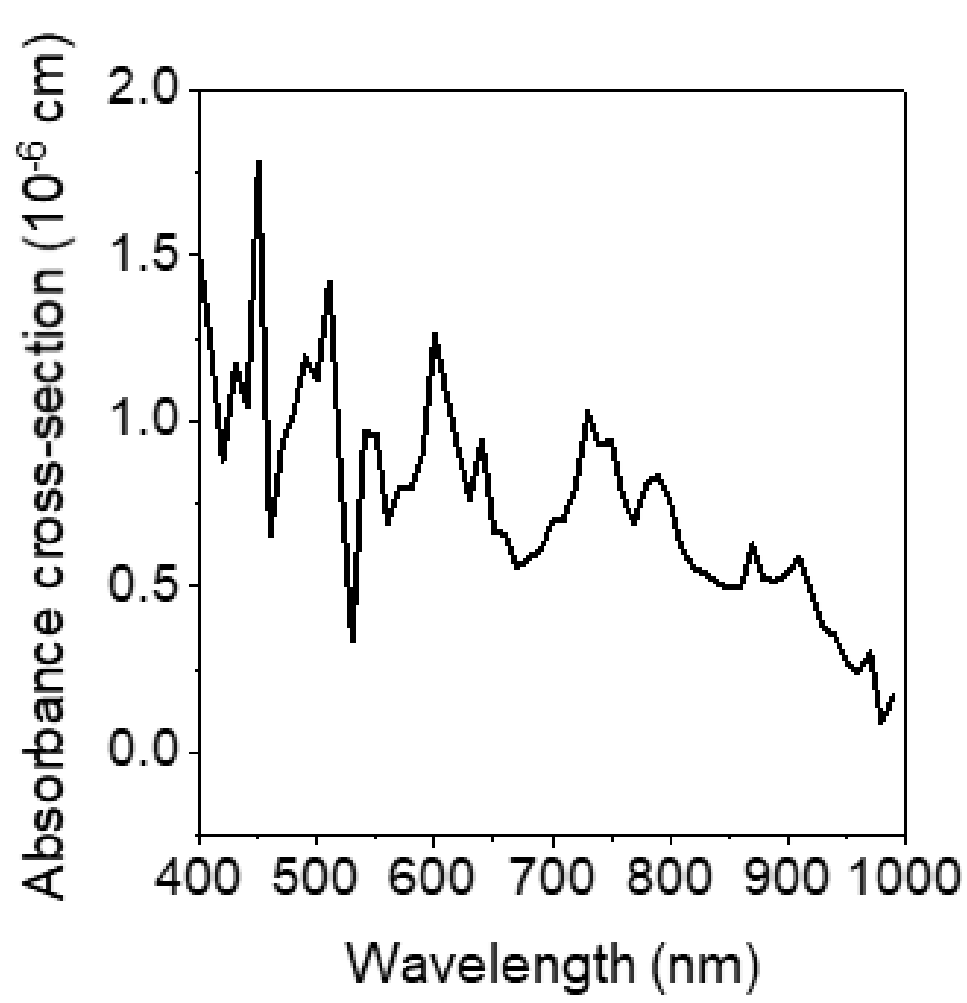

Figure S15. Simulated absorption cross-section of the nanogap as a function of wavelength, comparable to its physical cross-sectional area, indicating efficient optical confinement within the nanoscale channel.

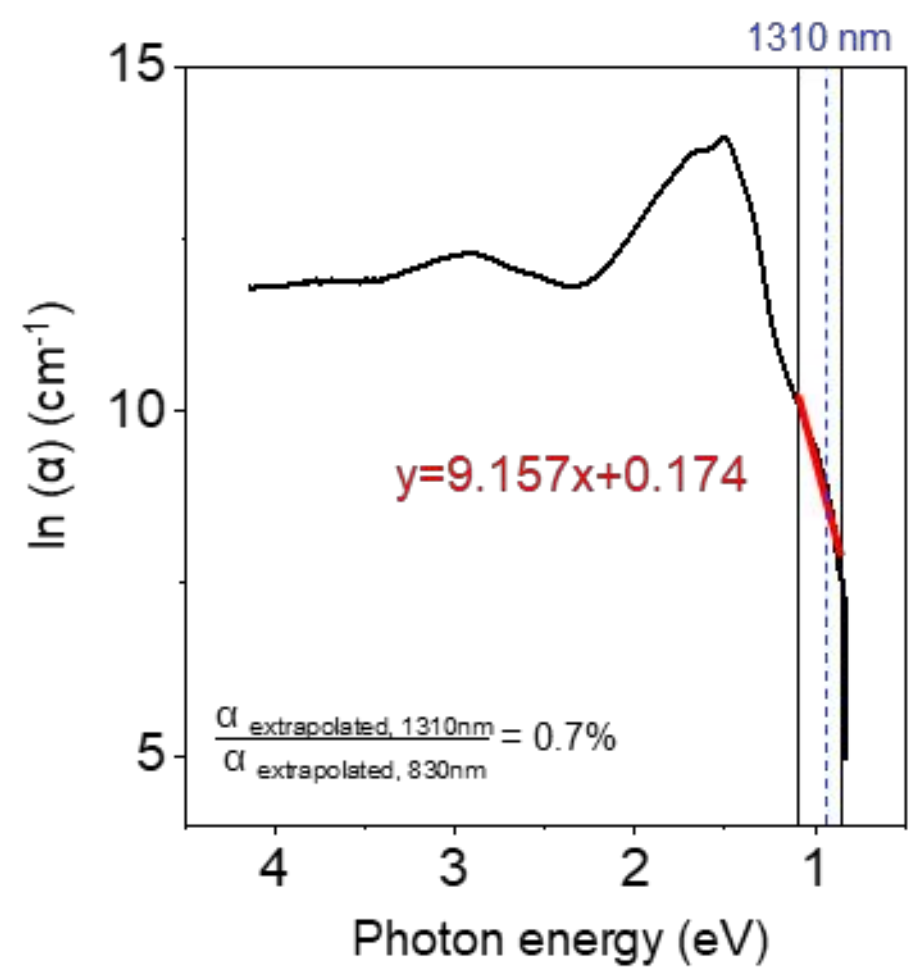

Figure S16. Urbach analysis of the absorption edge. The linear fitting of the exponential absorption tail was used to extrapolate the absorption coefficients at 830 and 1310 nm. The extrapolated absorption coefficient at 1310 nm is substantially lower than that at 830 nm, indicating a negligible contribution of direct BHJ absorption to the 1310 nm photoresponse.

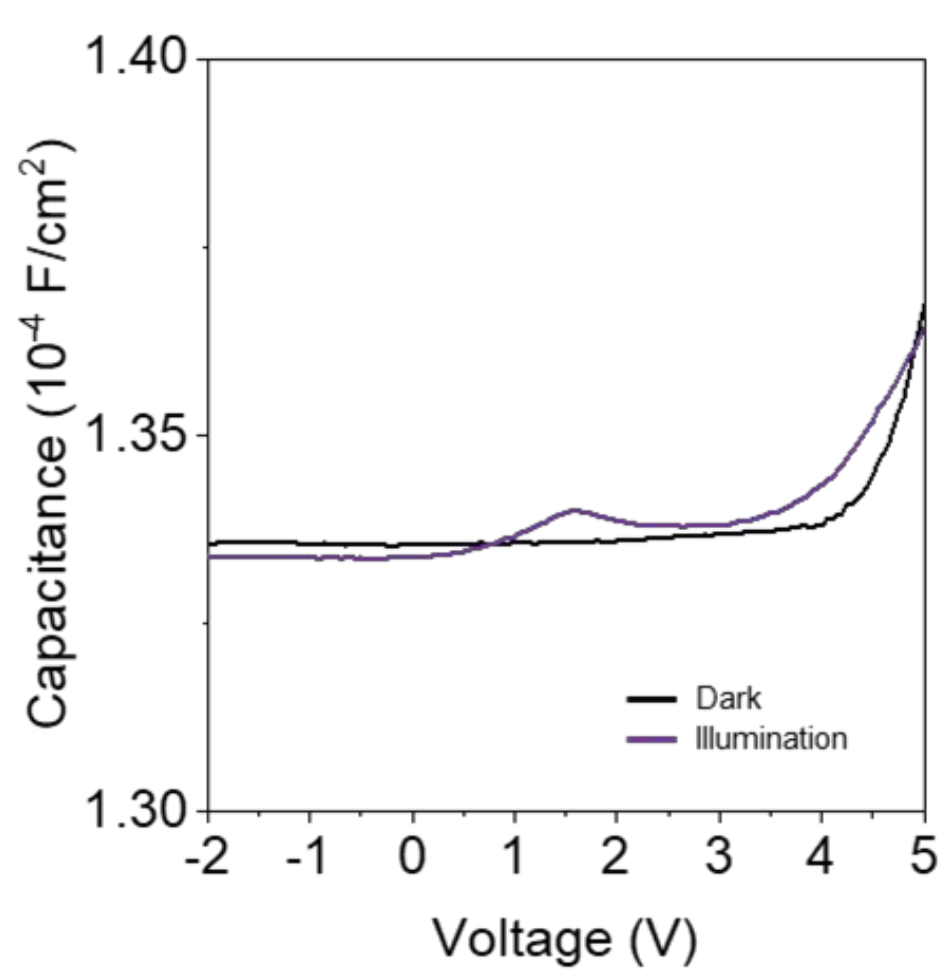

Figure S17. Capacitance–voltage (C–V) characteristics measured at 1310 nm, showing a shoulder peak at 1.5 V.

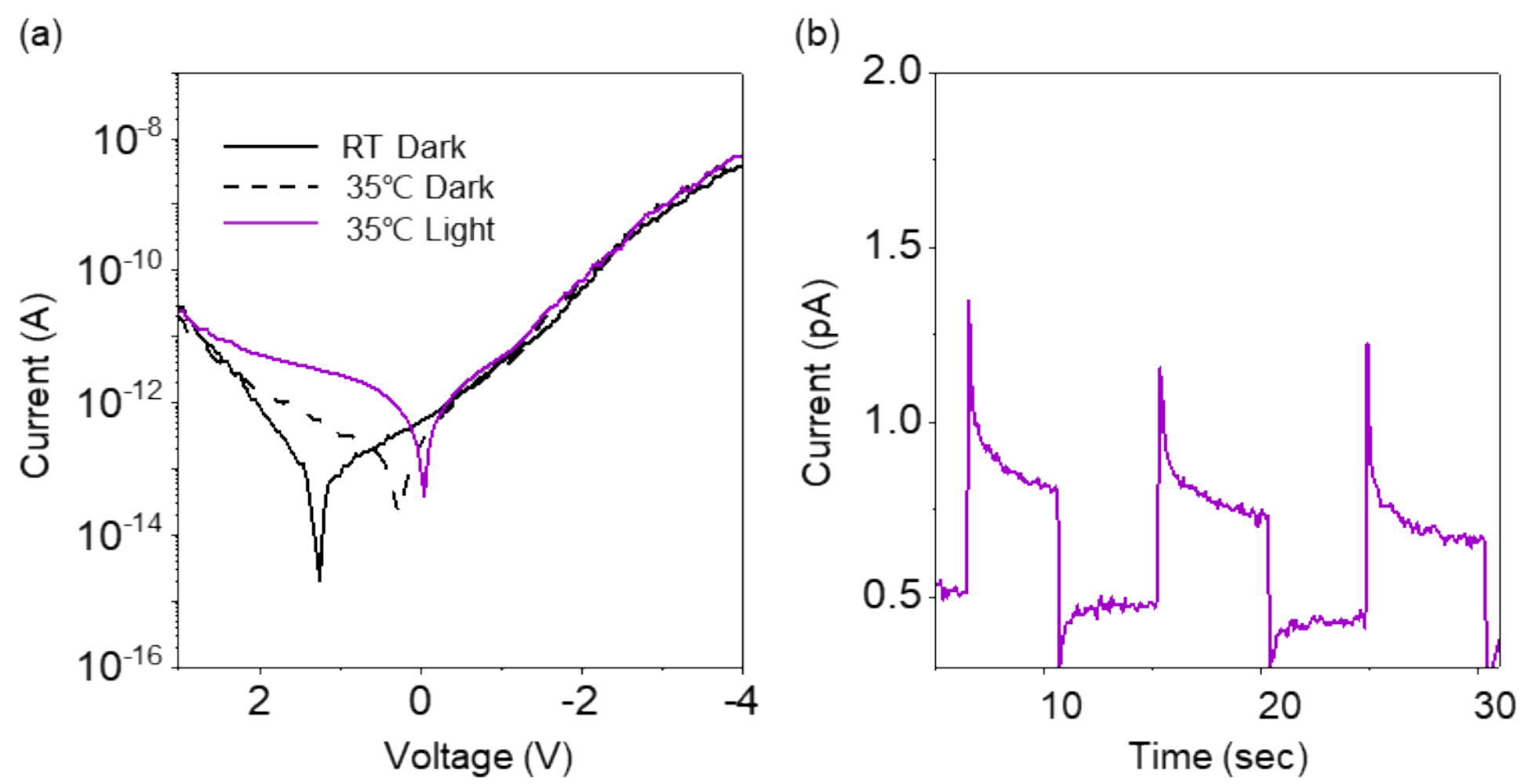

Figure S18. Temperature-dependent photoresponse characteristics of the PN-OPD. (a) I–V characteristics measured at room temperature and 35 ˚C, together with the illuminated I–V characteristics measured under 1310 nm illumination at 35 ˚C. (b) Time-dependent photocurrent measured at 35 ˚C under periodic 1310 nm illumination.

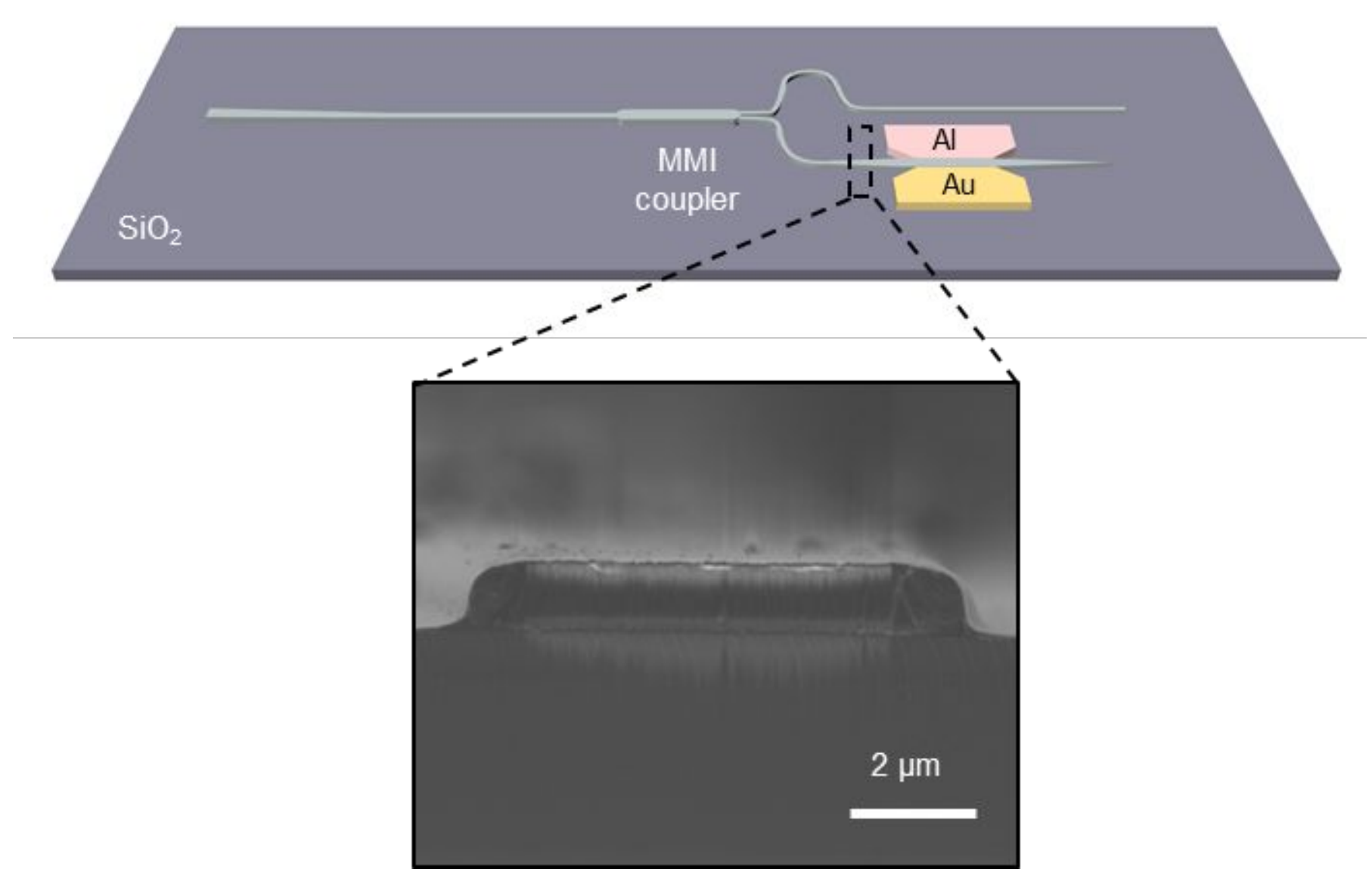

Figure S19. SEM image of the secondary main waveguide structure, showing a flat surface profile with a height of 1.2 μm.

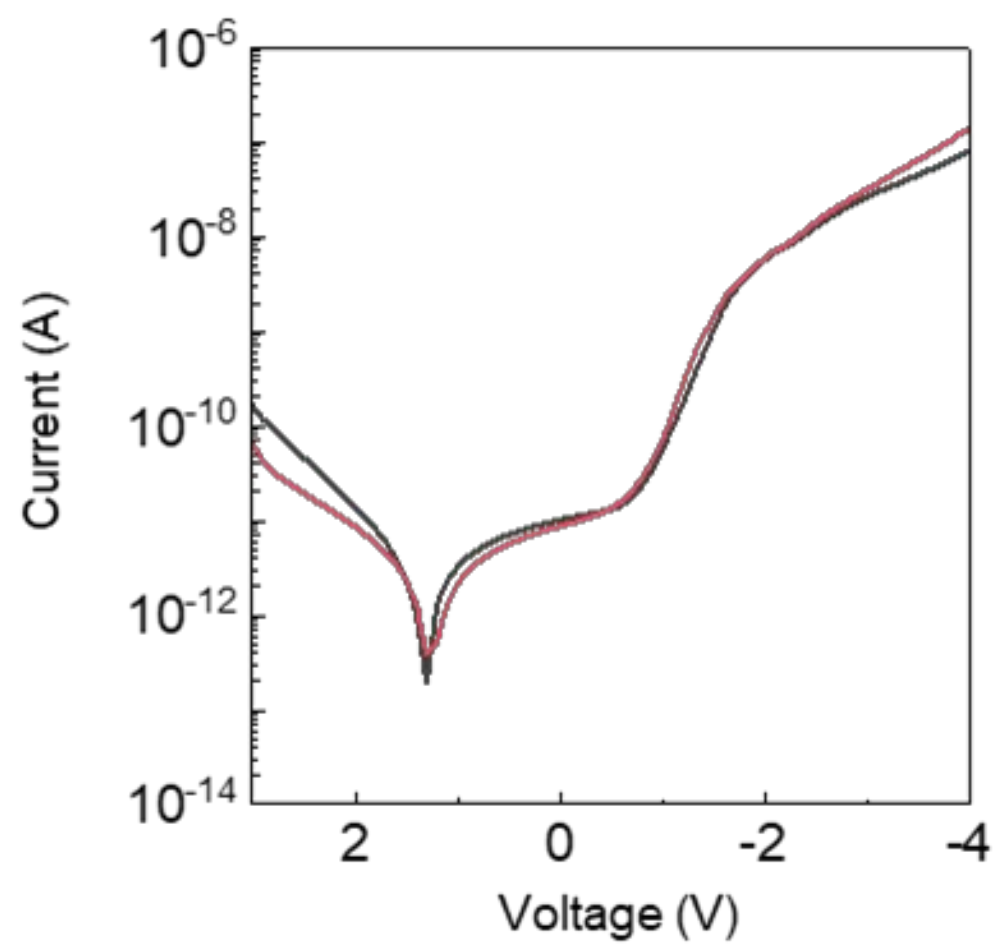

Figure S20. I–V characteristics measured before (black) and after (red) polymer waveguide integration, showing negligible changes in the electrical characteristics of the device.

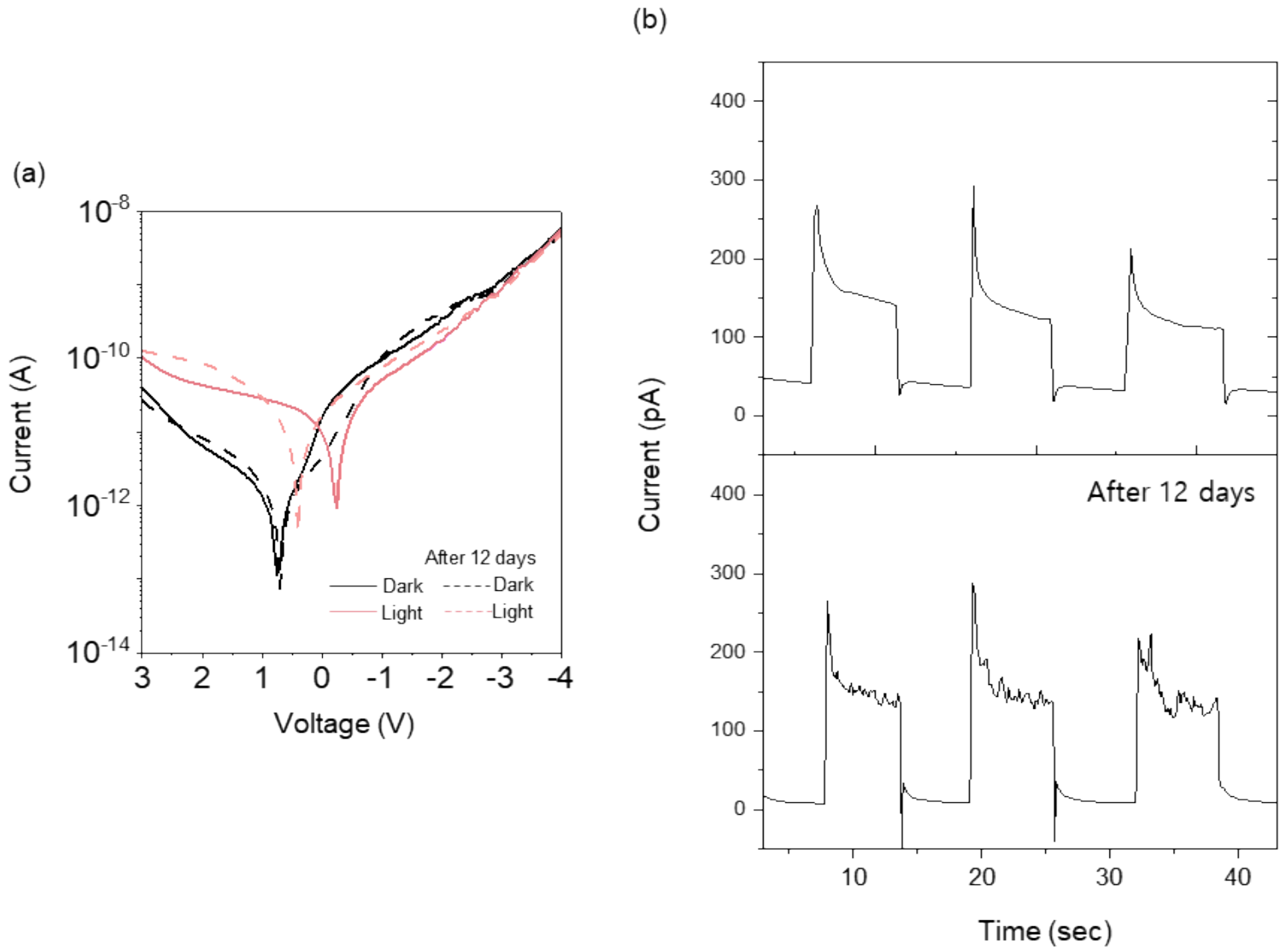

Figure S21. Stability evaluation of the PN-OPD. All measurements were performed under 830 nm illumination at an intensity of 1 mW cm⁻². (a) Dark and illuminated I–V characteristics measured before and after 12 days of storage under ambient conditions. (b) Time-dependent photocurrent measured under free-space illumination.

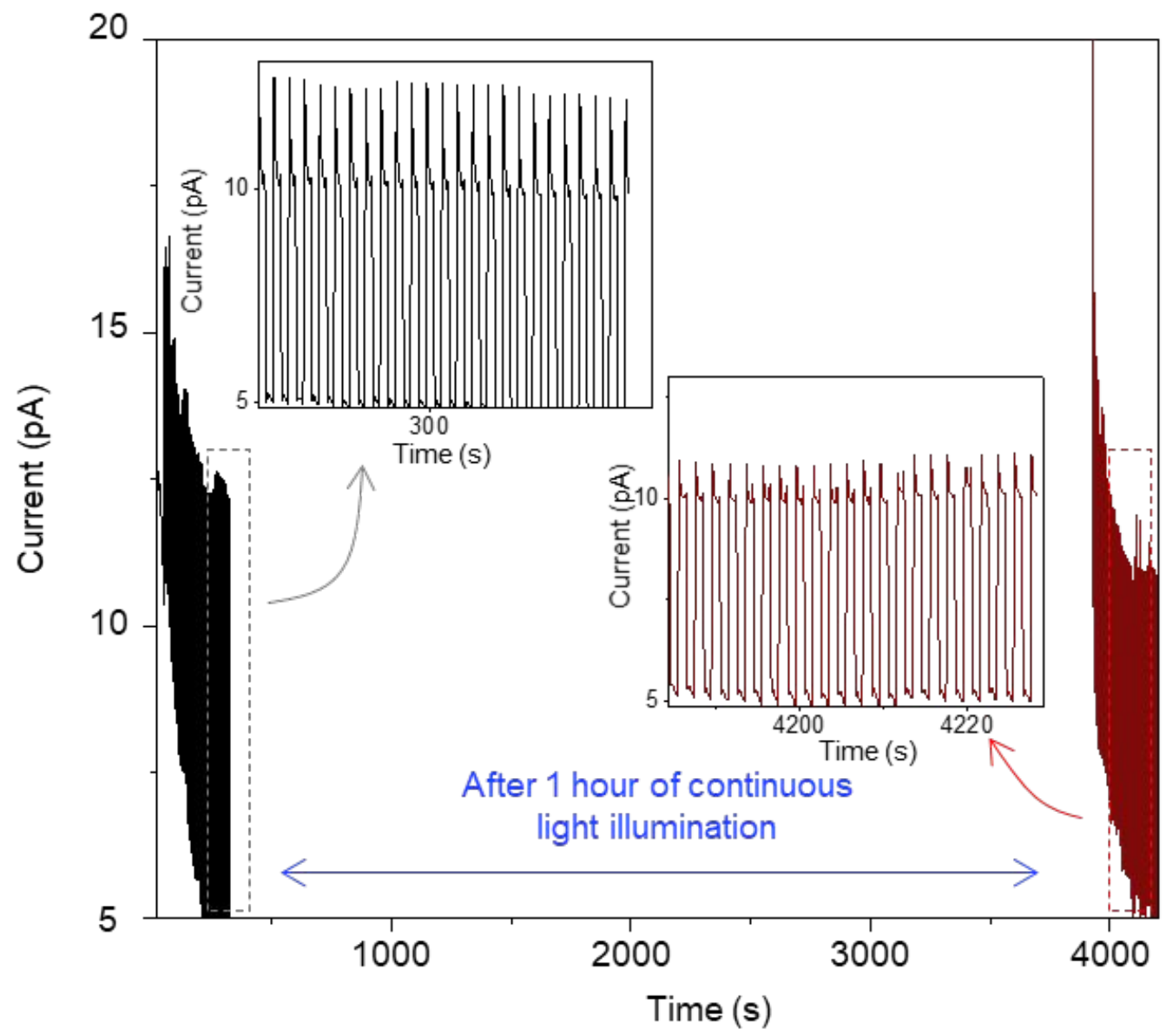

Figure S22. Continuous illumination stability of the PN-OPD. The transient photoresponse was measured under 830 nm illumination (1 mW cm^{-2}) with 1 Hz light modulation for 5 min before and after 1 h of continuous free-space illumination..

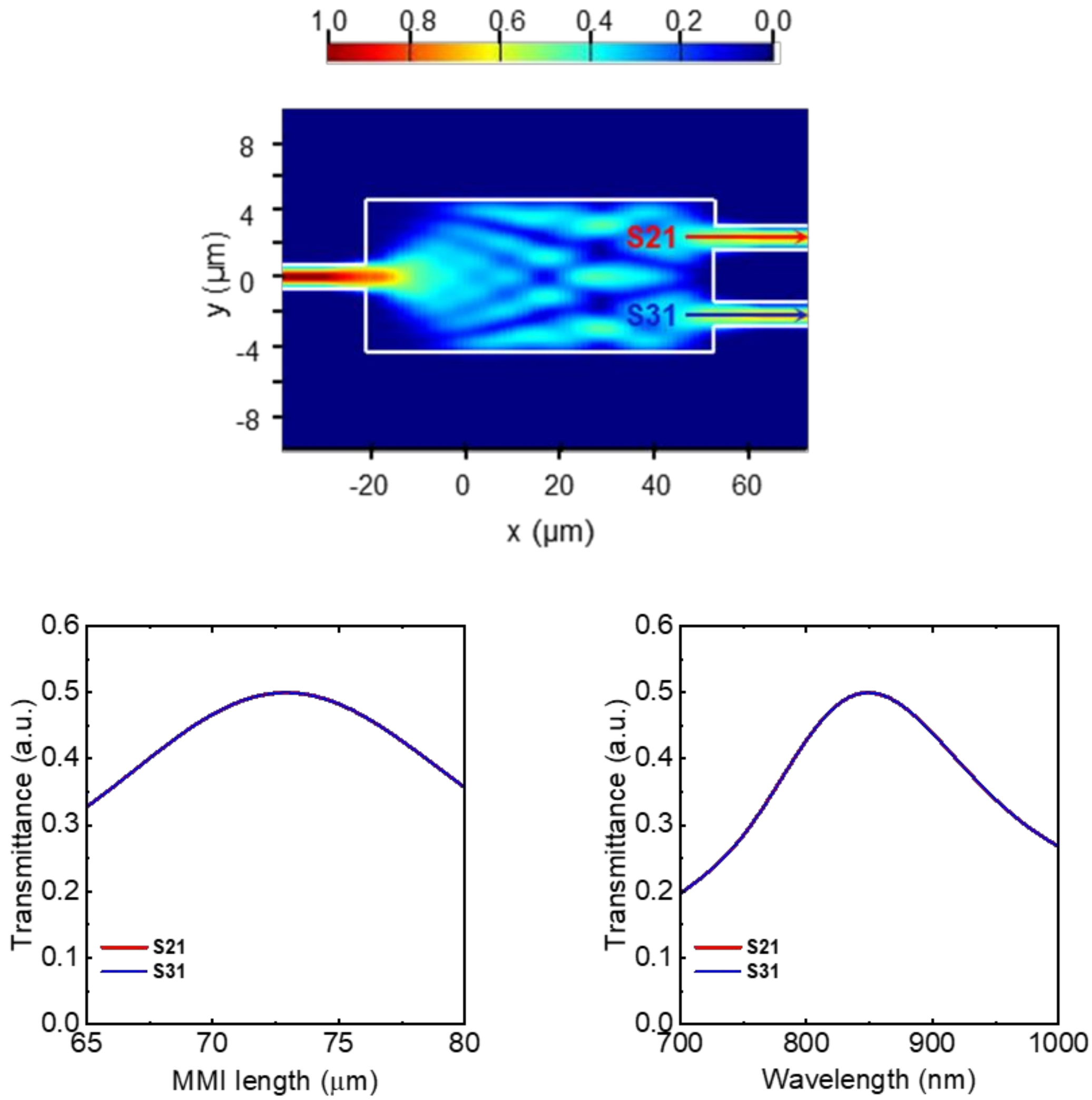

Figure S23. (Left) Simulated transmittance of the MMI structure as a function of device length, used to determine the optimal length for self-imaging. (Right) At the optimized length of 73 μm, the calculated S-parameters (S21 and S31) exhibit nearly equal transmission, confirming 50:50 power splitting into the two output waveguides.

Table R1. Summary of performance metrics of NIR photomultiplication-type organic photodetectors.

Wavelength (nm)	Bias (V)	EQE (%)	R (A/W)	Dark current density (A/cm^2)	Band-width (kHz)	D (Jones)	Rise time /Fall time	Active area (mm^2)	Waveguide integration$^+$	Ref.
830	3	6200	-	$\sim 10^{-7}$	7.2	5×10^{12}	-/45.4 μs	-	X	1
820	20	19.8*	-	-	0.0112	1.7×10^{12}	81.3ms /172.9 ms	-	X	2
843	-10	2000	-	10^{-5}	19.5	7×10^{11}	18 μs/667 μs	6.44	X	3
780	-10	600	4	3.6×10^{-7}	39	10^{13}	-	6.44	X	4
1050	1	3484	1	-	1.05	1.13×10^{12}	2.37 ms /0.58 ms (@1V)	-	X	5
800	20	370	2.38	$\sim 10^{-6}$	1	10^{9}	ms scale		X	6
840/1100	-10	1129/31	7.6/ 0.26	2.7×10^{-4}	43	1.5×10^{10}/ 5.1×10^{8}	73/76 μs	4.8	X	7
830	1	500	3	10^{-5}	**354**	6×10^{9}	11.3 μs /26.2 μs	3×10^{-6}	O	**This work**

$^+$ Waveguide integration indicates whether waveguide-coupled photodetection was experimentally demonstrated in the cited work.

Table S2. Summary of performance metrics of waveguide-integrated photodetectors.

Active material		Device architecture	Wavelength (nm)	R (A/W)	Band width	Dark current (@ V)	Ref.
Inorganic PD	InGaAs	Hybrid III–V/Si PIN photodiode	1550	0.36	30 GHz	-	8
	Graphene	Twisted bilayer PD	1550	0.65	65 GHz	140 μA (0.5 V)	9
	Graphene	Plasmonic PD	1550	12.2 V/W	42 GHz	-	10
	Ge	PIN	1510	0.2	-	0.1 μA (-2 V)	11
	Si	Lateral PIN PD	850	0.44	14 GHz	0.2 μA (-25V)	12
2D PD	MoS_2	Hot electron-assisted Schottky PD	1550	1.57	1.37 GHz	1 μA (-0.3 V)	13
	$MoTe_2$ /Graphene	Vertical van der Waals heterostructure	1300	0.2	24 GHz	~3 μA (0 V)	14
	$MoSe_2$ /WS_2	Heterojunction	780	1	20 MHz	5×10^{-5} μA (2V)	15
Colloidal quantum dot PD	PbS	Vertical PIN PD	2100	1.3	1.1 MHz	0.1 μA (-3V)	16
Organic PD	CuPc :PTCBI	BHJ PD	650	-	-	-	17
	ZnPC:C60	BHJ PD	635	-	-	10^{-3} μA (-0.5V)	18
	DPPTT :IEICO-4Cl	PN-OPD	830	**160**	354 kHz	10^{-6} μA (1V)	**This work**

Supplemetary References

(1) Han, S. G.; Lee, H.; Choi, W.; Lee, D.; Kim, S.; Sung, Y.; Kim, S.; Cho, K. Photomultiplication-type organic photodetectors with fast response enabled by the controlled charge trapping dynamics of quantum dot interlayer. *Advanced Functional Materials* **2021**, *31* (31), 2102087.

(2) Shou, M.; Zhang, Q.; Xiong, S.; Han, T.; Zhou, J.; Zheng, N.; Xie, Z.; Liu, L. Simultaneous photomultiplication improvement and response acceleration for high-performance lateral polymer photodetectors based on bulk heterojunction. *ACS applied materials & interfaces* **2021**, *13* (4), 5293-5301.

(3) Kublitski, J.; Fischer, A.; Xing, S.; Baisinger, L.; Bittrich, E.; Spoltore, D.; Benduhn, J.; Vandewal, K.; Leo, K. Enhancing sub-bandgap external quantum efficiency by photomultiplication for narrowband organic near-infrared photodetectors. *Nature Communications* **2021**, *12* (1), 4259.

(4) Xing, S.; Kublitski, J.; Hänisch, C.; Winkler, L. C.; Li, T. y.; Kleemann, H.; Benduhn, J.; Leo, K. Photomultiplication-type organic photodetectors for near-infrared sensing with high and bias-independent specific detectivity. *Advanced Science* **2022**, *9* (7), 2105113.

(5) Huang, Y.; Shao, L.; Wang, Y.; Hao, L.; Luo, X.; Zheng, J.; Cao, Y.; Li, S.; Tan, Z.; Li, S. Bias-Switchable Photomultiplication and Photovoltaic Dual-Mode Near-Infrared Organic Photodetector. *Advanced Materials* **2025**, *37* (15), 2500491.

(6) Hu, X.; Li, N.; Ren, M.; Ji, Y.; Guo, H.; Chen, Q.; Sui, X. Photovoltaic and Photomultiplication Dual-Mode Near-Infrared Organic Detectors with Large Dynamic Range for Intensive and Faint Light Sensing. *Advanced Science* **2025**, *12* (32), e06499. DOI: https://doi.org/10.1002/advs.202506499.

(7) Brunetta, M.; Qiao, Z.; Nodari, D.; Sasaki, K.; Furlan, F.; Rimmele, M.; Kim, H.; Chen, H.; Tagawa, Y.; Marsh, A.; et al. Selective Charge Extraction Allows Fast Photomultiplication and Imaging in Rigid and Ultra-Flexible SWIR Organic Photodiodes. *Advanced Energy Materials* **2026**, *16* (16), e06776. DOI: https://doi.org/10.1002/aenm.202506776 (acccessed 2026/08/18).

(8) Piels, M.; Bauters, J. F.; Davenport, M. L.; Heck, M. J. R.; Bowers, J. E. Low-Loss Silicon Nitride AWG Demultiplexer Heterogeneously Integrated With Hybrid III–V/Silicon Photodetectors. *J. Lightwave Technol.* **2014**, *32* (4), 817-823.

(9) Wu, Q.; Qian, J.; Wang, Y.; Xing, L.; Wei, Z.; Gao, X.; Li, Y.; Liu, Z.; Liu, H.; Shu, H.; et al. Waveguide-integrated twisted bilayer graphene photodetectors. *Nature Communications* **2024**, *15* (1), 3688. DOI: 10.1038/s41467-024-47925-x.

(10) Muench, J. E.; Ruocco, A.; Giambra, M. A.; Miseikis, V.; Zhang, D.; Wang, J.; Watson, H. F. Y.; Park, G. C.; Akhavan, S.; Sorianello, V.; et al. Waveguide-Integrated, Plasmonic Enhanced Graphene Photodetectors. *Nano Letters* **2019**, *19* (11), 7632-7644. DOI: 10.1021/acs.nanolett.9b02238.

(11) Kang, J.; Takagi, S.; Takenaka, M. Ge photodetector monolithically integrated with amorphous Si waveguide on wafer-bonded Ge-on-insulator substrate. *Opt. Express* **2018**, *26* (23), 30546-30555. DOI: 10.1364/OE.26.030546.

(12) Chatterjee, A.; Saumitra; Sikdar, S. K.; Selvaraja, S. K. High-speed waveguide integrated silicon photodetector on a SiN-SOI platform for short reach datacom. *Opt. Lett.* **2019**, *44* (7), 1682-1685. DOI: 10.1364/OL.44.001682.

(13) Li, Z.; Hu, S.; Zhang, Q.; Tian, R.; Gu, L.; Zhu, Y.; Yuan, Q.; Yi, R.; Li, C.; Liu, Y.; et al. Telecom-Band Waveguide-Integrated MoS2 Photodetector Assisted by Hot Electrons. *ACS Photonics* **2022**, *9* (1), 282-289. DOI: 10.1021/acsphotonics.1c01622.

(14) Flöry, N.; Ma, P.; Salamin, Y.; Emboras, A.; Taniguchi, T.; Watanabe, K.; Leuthold, J.; Novotny, L. Waveguide-integrated van der Waals heterostructure photodetector at telecom wavelengths with high speed and high responsivity. *Nature Nanotechnology* **2020**, *15* (2), 118-124. DOI: 10.1038/s41565-019-0602-z.

(15) Gherabli, R.; Indukuri, S. R. K. C.; Zektzer, R.; Frydendahl, C.; Levy, U. MoSe2/WS2 heterojunction photodiode integrated with a silicon nitride waveguide for near infrared light detection with high responsivity. *Light: Science & Applications* **2023**, *12* (1), 60. DOI: 10.1038/s41377-023-01088-4.

(16) Pang, C.; Deng, Y.-h.; Kheradmand, E.; Hagelsieb, L. M.; Guo, Y.; Cheyns, D.; Geiregat, P.; Hens, Z.; Van Thourhout, D. A silicon photonics waveguide-coupled colloidal quantum dot photodiode sensitive beyond 1.6 µm. *APL Photonics* **2024**, *9* (6), 066113. DOI: 10.1063/5.0206386 (acccessed 7/16/2026).

(17) Bernhard, L.; Martin, S.; Elke, K.; Paul, H.; Georg, J.; Stefan, K.; Harald, D.; Nicole, G.; Joachim, K.; Birgit, U.; et al. Integrated waveguide sensor platform utilizing organic photodiodes. In *Proc.SPIE*, 2012; Vol. 8234, p 82341H. DOI: 10.1117/12.908432.

(18) Otto, T.; Rabe, T.; Döring, S.; Kowalsky, W. Organic Solar Cells as Polymer Waveguide Integrated Photodetectors. *Procedia Technology* **2014**, *15*, 156-160. DOI: https://doi.org/10.1016/j.protcy.2014.09.067.
